# Supplementary material for: Biodereplication of Antiplasmodial Extracts: Application of the Amazonian Medicinal Plant Piper coruscans Kunth
Source: Molecules. 2022 Nov 7;27(21):7638. doi: 10.3390/molecules27217638 (PMC9656727; doi:10.3390/molecules27217638)
Supplement: Supplementary file 1 [file molecules-27-07638-s001.zip › molecules-1985904-supplementary.pdf]

Table of Content

SI 1. HPLC-DAD chromatogram of alkaloid extract from *Cinchona pubescens* S3

SI 2. ESI (+) HRMS spectra of antimalarial drugs with heme-Fe(III) S4

SI 3. ESI (+) HRMS spectrum of artemisinin-Fe(II) heme S8

SI 4. Molecular network of ethyl acetate extract from *Piper coruscans* S9

SI 5. Experimental S10

SI 6. CPC fractograms of cyclohexane extract from *P. coruscans* S14

SI 7. Proton NMR of known compounds isolated for *P. coruscans* S17

SI 8. 1D and 2D NMR for compound 10 S28

SI 9. NMR spectroscopic data for the isolated compounds S33

SI 10. LCMS of compounds isolated from *P. coruscans* *S*36

SI 11. Structure of antimalarial drugs SI38

SI 12. Structure of alkaloids from *Cinchona pubescens* SI39

SI 13. Reference

SI 1. HPLC-DAD (280 nm) chromatogram of alkaloid extract from *Cinchona pubescens*

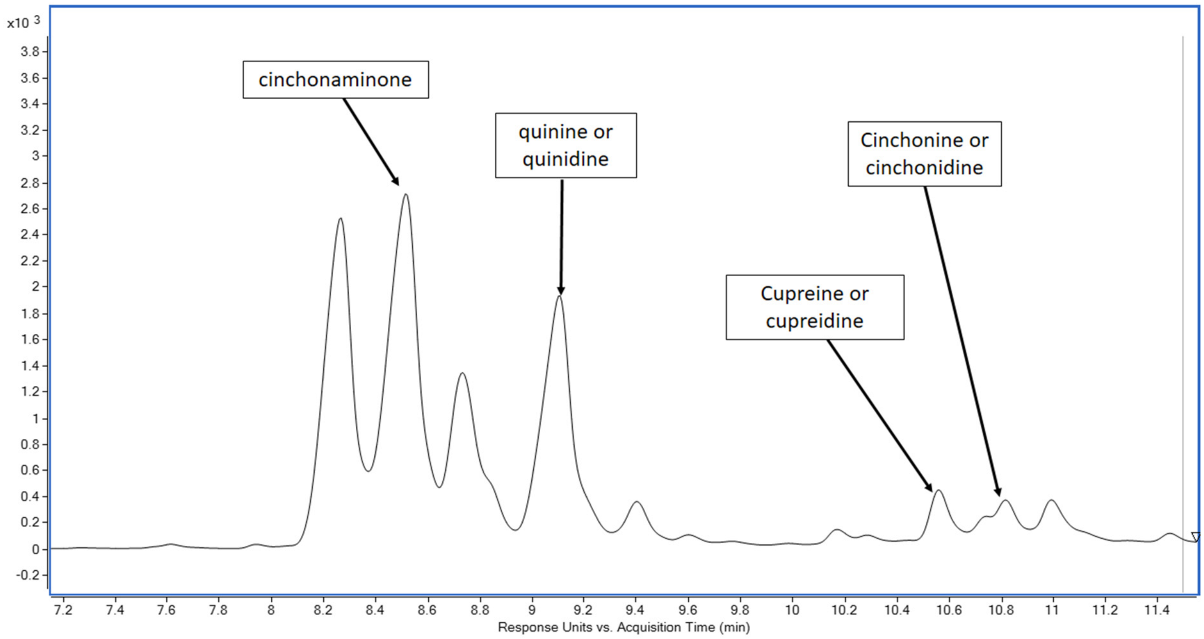

Figure S1 HPLC-DAD (280 nm) chromatogram of alkaloid extract from Cinchona pubescens

SI 2. ESI (+) HRMS spectra of antimalarial drugs with heme-Fe(III)

Figure S2. ESI (+) HRMS spectra of antimalarial drugs with heme-Fe(III)

ESI (+) spectrum of mixture chloroquine diphosphate - heme

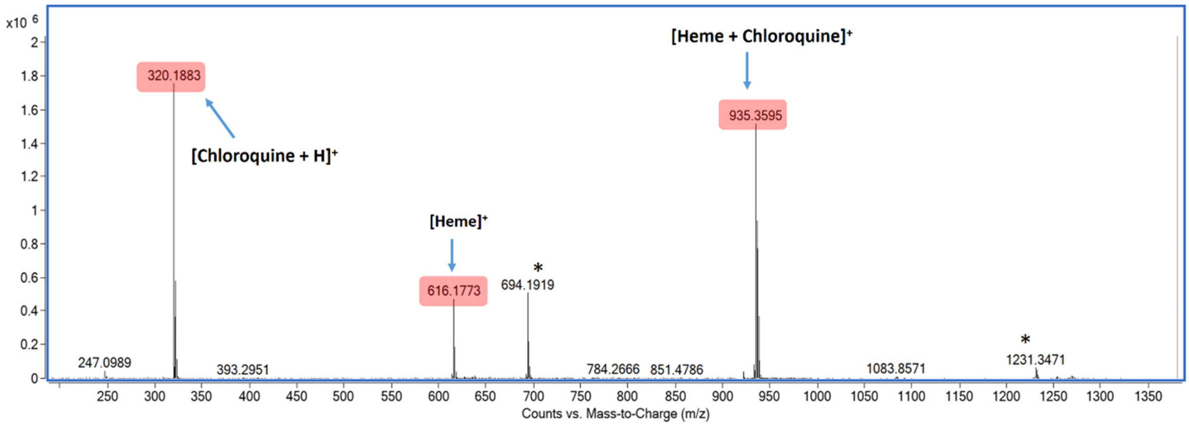

ESI (+) spectrum of mixture quinine - heme

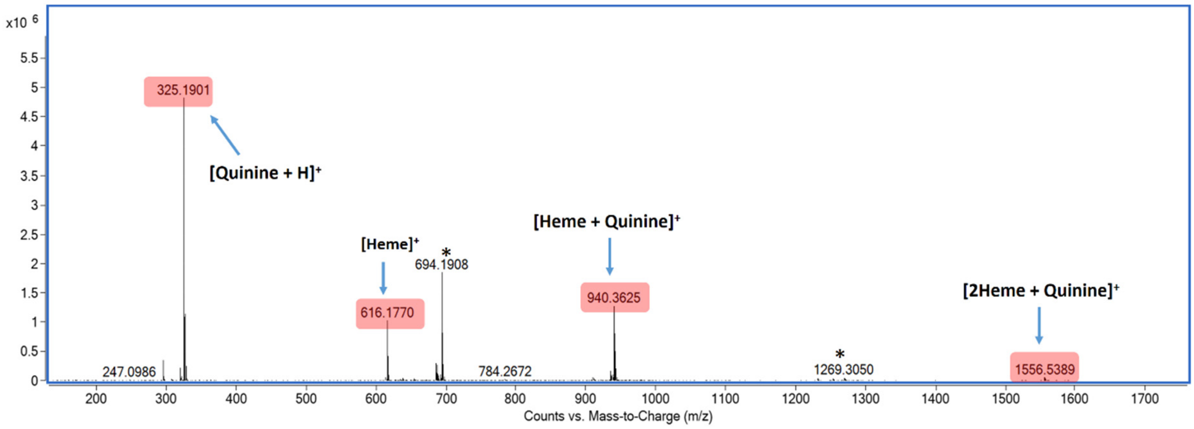

ESI (+) spectrum of mixture amodiaquine - heme

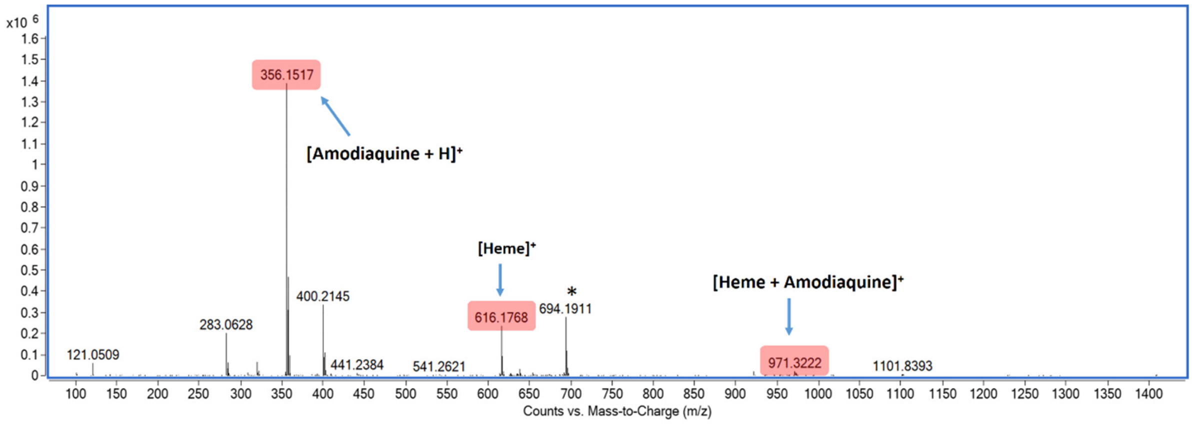

ESI (+) spectrum of mixture ketoconazole - heme

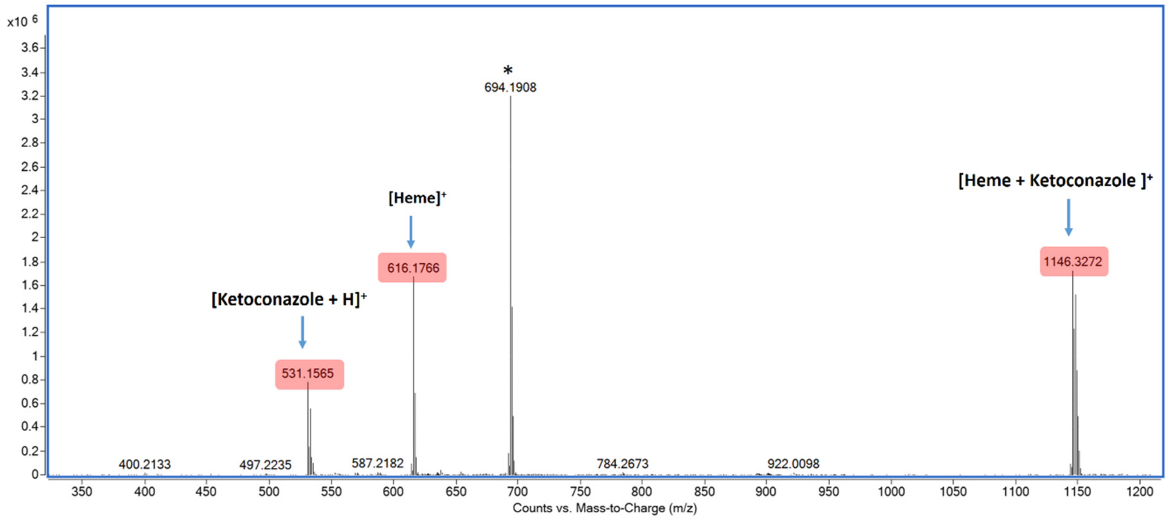

ESI (+) spectrum of mixture mefloquine - heme

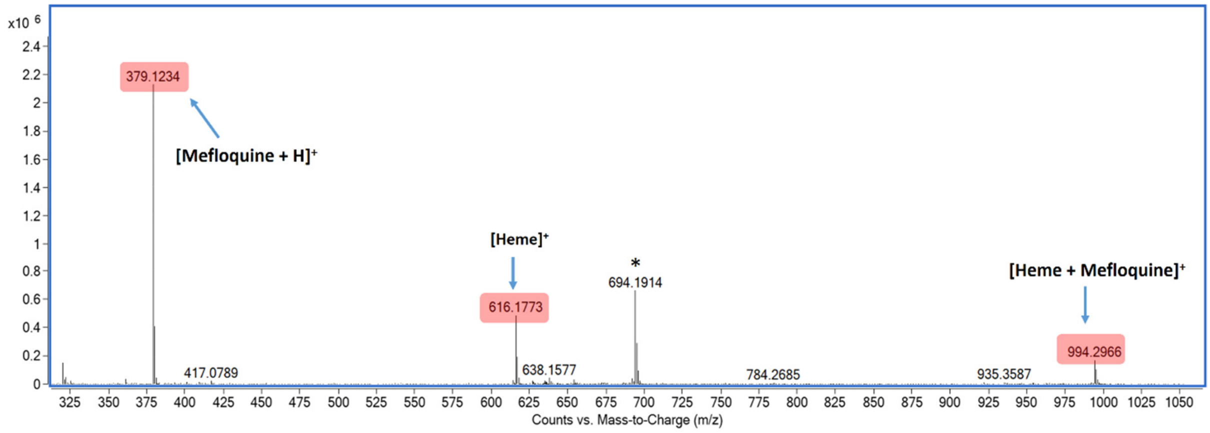

ESI (+) spectrum of sulfadoxine - heme

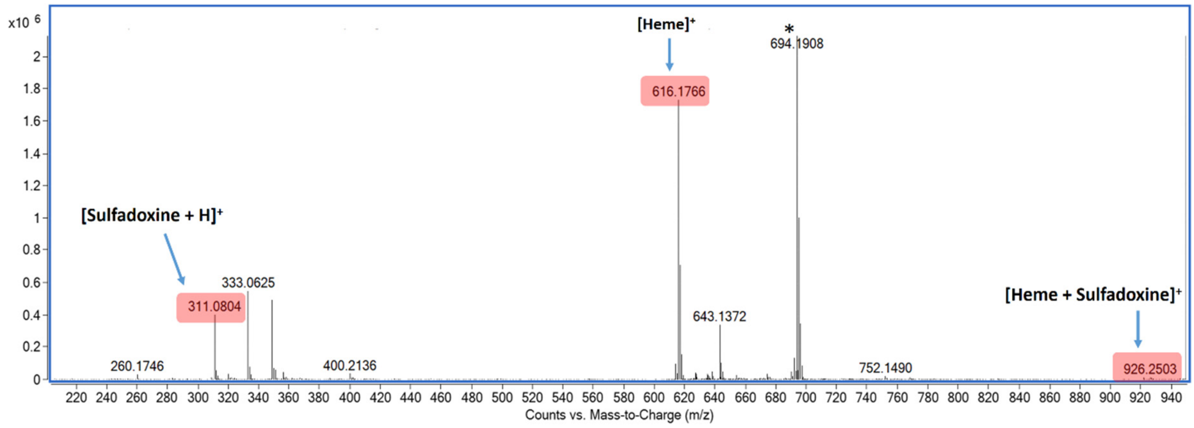

ESI (+) spectrum of mixture praziquantel - heme

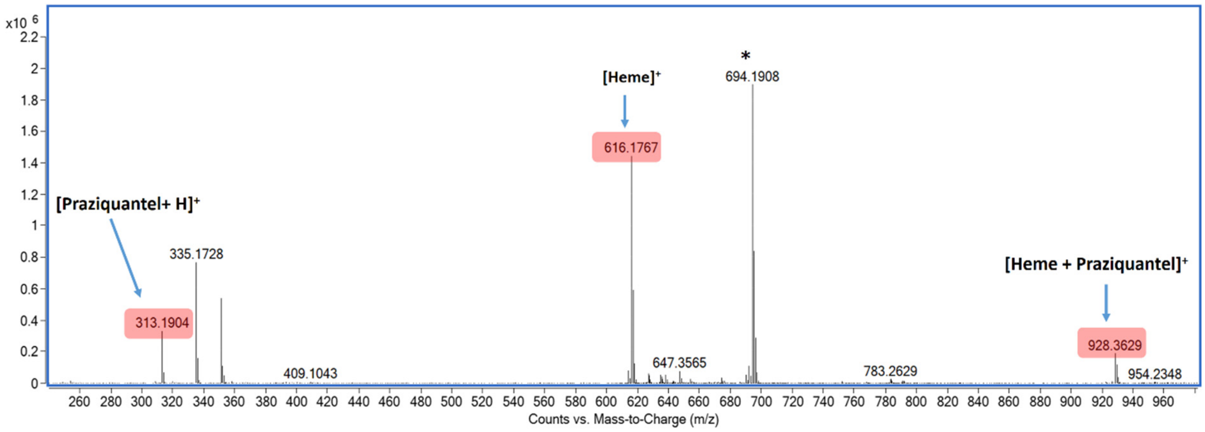

ESI (+) spectrum of mixture miconazole - heme

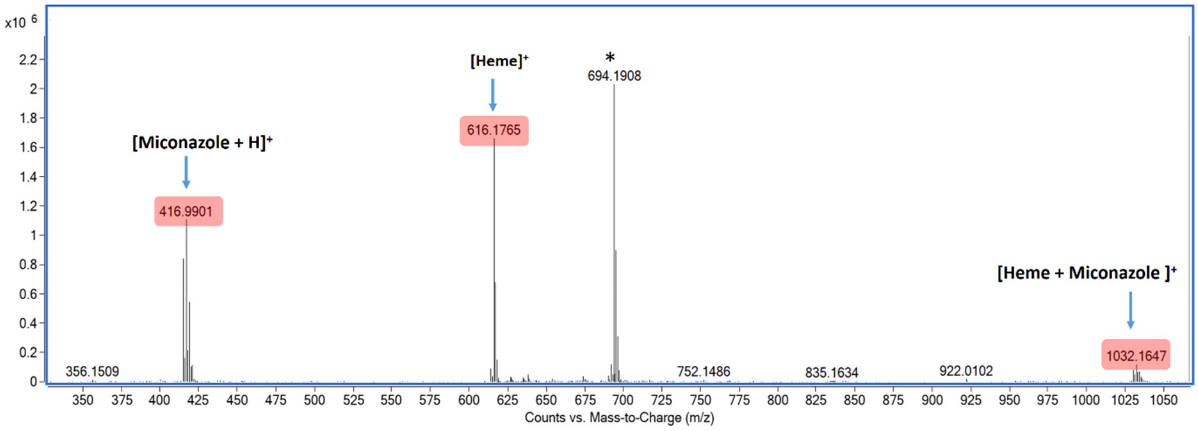

SI 3. ESI (+) HRMS spectrum of artemisinin - Fe(II) heme

Figure S3. ESI (+) HRMS spectrum of artemisinin - Fe(II) heme

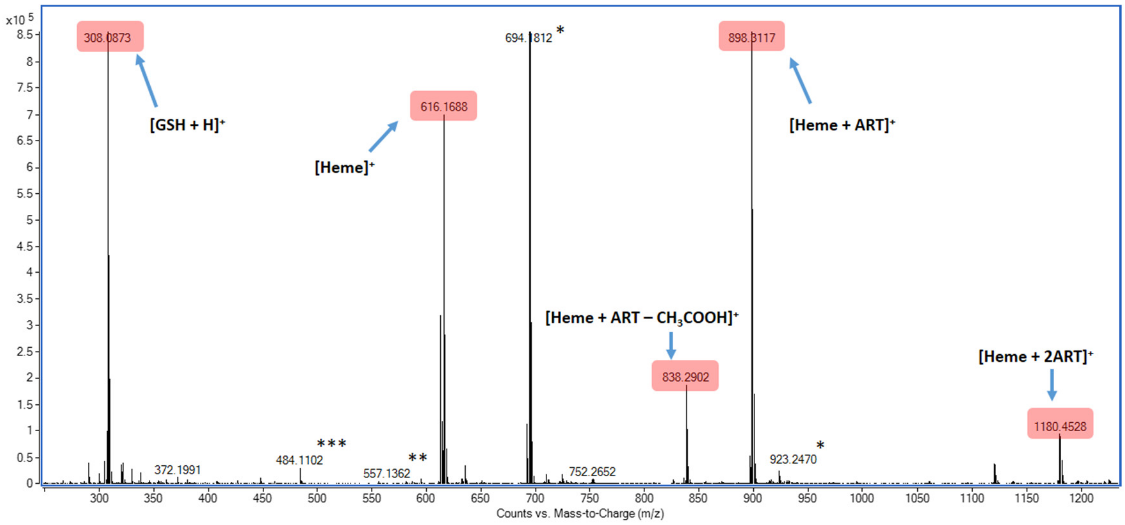

*Heme adducts with DMSO, GSH or heme dimers ([2heme – H]+, [2heme + 37Cl]+); ** fragment of heme following loss of CH_2-COOH; *** fragment GSSG following loss of γ-glutamate.

SI 4. Molecular network of ethyl acetate extract from *Piper coruscans*

Figure S4. Molecular network of ethyl acetate extract from *Piper coruscans*

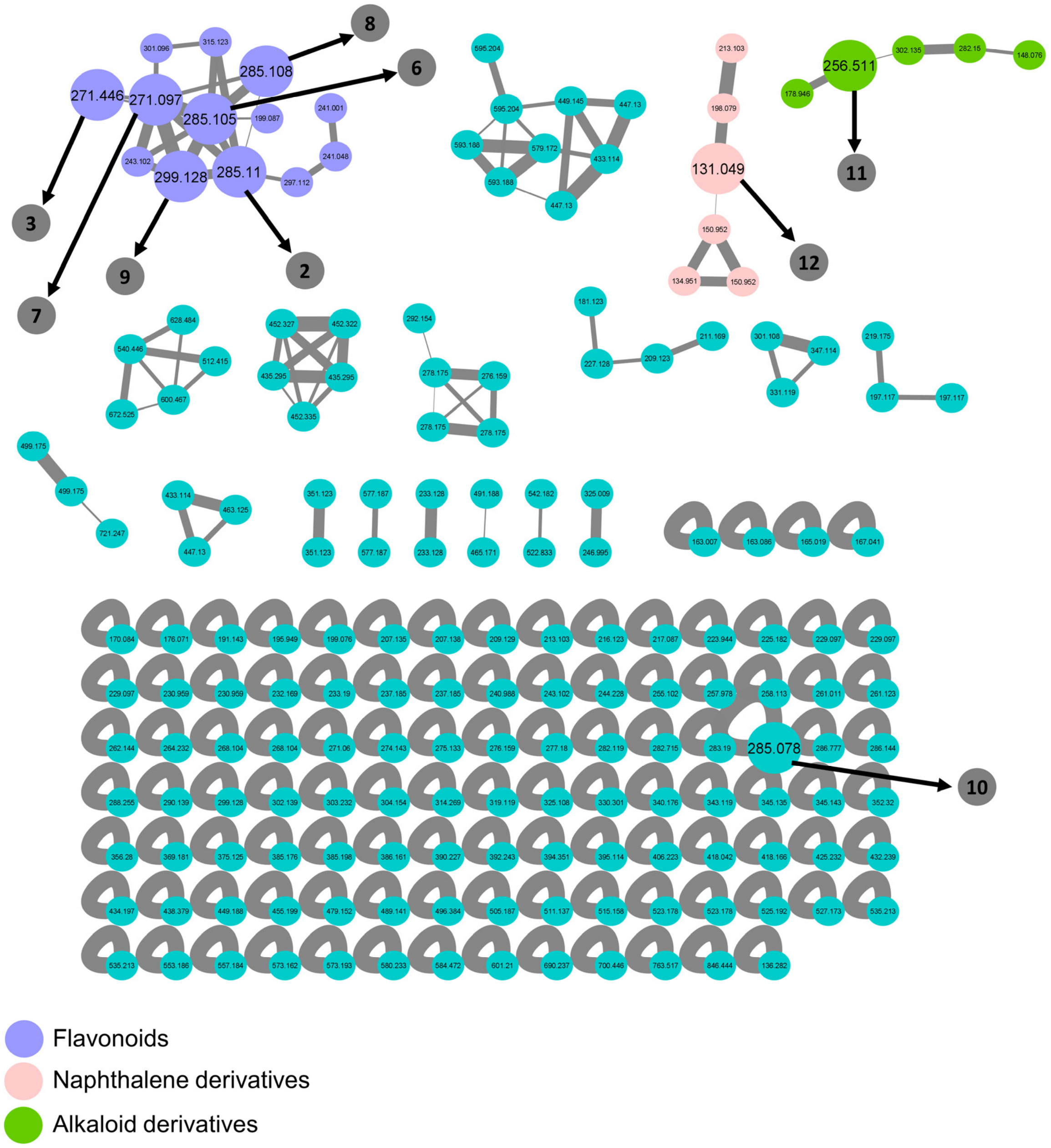

SI 5. Experimental

Materials

Antimalarial drugs, reagents and solvents were acquired from Sigma-Aldrich (France) or other providers when specified: chloroquine diphosphate salt, quinine chloride, amodiaquine dihydrochloride dehydrate, miconazole nitrate salt, ketoconazole, sulfadoxine ≥ 95 % (TLC), praziquantel, mefloquine hydrochloride and artemisinin, Tween 20, citric acid, ACS reagent, ≥99.5%, LCMS-grade acetonitrile, LCMS-grade methanol, n-octanol for analysis (Carlo ERBA reagents, France), n-heptane for analyses (Scharlau), ethyl acetate for analyses (VWR chemical), n-butanol for analyses (EMSURE® ACS,ISO), methanol for analyses (EMSURE® ACS,ISO), water Milli-Q. NMR of compounds was carried in deutered solvents ($CDCl_3$, CD_3CN or MeOD). Deionized water was obtained from a Milli-Q instrument (Millipore, France).

Extraction of alkaloidic extract from *Cinchona pubescens* Vahl (Rubiaceae) was carried in December 2011 in the Pharmacognosy laboratory at Paris-Saclay University (France), as described in Durango *et al* [1].

Known compounds were identified based on literature spectral data. Aurentiacin (**1**) [2] ; stercurensin, (**2**) [3,4]; cardamomin, (**3**) [4,5]; strobopinin 7-methyl ether (**4**) [6]; 5-hydroxy-7-methoxy-6,8-dimethyl flavanone (**5**) [7]; desmethoxymatteucinol (**6**) [8]; alpinetin, (**7**) [4,9]; pinocembrin, (**8**) [10]; dimethyl cryptostrobin, (**9**) [11]; *N*-Benzoyltyrarnine methyl ether, (**11**) [12,13]; 1*H*-inden-1-one (**12**) [14,15].

Analyses

$[\alpha]_D$ analyses were carried used Atago™ Polarimetre Polax-2L carried at λ = 589 at a temperature of 16 °C. All compounds were yellow pale. For **4**, $[\alpha]_D$ + 7.02 (c 0.02 in MeOH); for **5**, $[\alpha]_D$ + 3.34 (c 0.01 in MeOH); for **6**, $[\alpha]_D$ + 3.51 (c 0.01 in MeOH); for **10**, $[\alpha]_D$ + 7.38 (c 0.02 in MeOH); for **9**, $[\alpha]_D$ + 7.02 (c 0.02 in MeOH); and for **11**, $[\alpha]_D$ + 10.03 (c 0.03 in MeOH).

Positive (+) HRMS $[M + H]^+$ was performed in 6530 Accurate-Mass QToF LC/MS instrument (Agilent Technologies). (+) HRMS $[M + H]^+$ for **1** *m/z* 299.1228 (calcd for $C_{18}H_{18}O_4$ 298.3153); (+) HRMS $[M + H]^+$ for **2** *m/z* 285.1101 (calcd for $C_{17}H_{16}O_4$ 284.1115); (+) HRMS $[M + H]^+$ for **3** *m/z* 271.0943 (calcd for $C_{16}H_{14}O_4$ 271.0930); (+) HRMS $[M + H]^+$ for **4** *m/z* 285.1109 (calcd for $C_{17}H_{16}O_4$ 284.1063); (+) HRMS $[M + H]^+$ for **5** *m/z* 299.1274 (calcd for $C_{18}H_{18}O_4$ 298.1253); (+) HRMS $[M + H]^+$ for **6** *m/z* 285.1101 (calcd for $C_{17}H_{16}O_4$ 284.1115); (+) HRMS $[M + H]^+$ for **7** *m/z* 271.4951 (calcd for $C_{16}H_{14}O_4$ 271.4958); (+) HRMS $[M + H]^+$ for **8** *m/z* 285.1088 (calcd for $C_{17}H_{16}O_4$ 284.1117); (+) HRMS $[M + H]^+$ for **9** *m/z* 299.1270

(calcd for $C_{18}H_{18}O_4$ 298.1271); (+) HRMS [M + H]$^+$ for **10** *m/z* 285.1109 (calcd for $C_{17}H_{16}O_4$ 285.1108); (+) HRMS [M + H]$^+$ for **11** *m/z* 256.1334 (calcd for $C_{16}H_{17}NO_2$ 255.1342); (+) HRMS [M + H]$^+$ for **12** *m/z* 131.0476 (calcd for C_9H_6O 131.0478).

NMR analyses were performed on a NMR Avance Bruker 500 MHz and 600 MHz CryoProbe for all compounds. Results were carried on Bruker TopSpin Software/NMR Data Analysis.

NMR analyses of compound 10 (ethyl 5-cinnamoyl-4,5-dihydroxy-3-methyl-4,5-dihydrofuran-2-carboxylate): Compound **10** was obtained as a yellow, amorphous powder; UV (MeOH), λmax = 280 nm. (+) HREMS indicated a [M + H]$^+$ ion peak at *m/z* 285.1109, consistent with the molecular formula $C_{17}H_{16}O_4$. This product is obtained through the spontaneous fragmentation of compound **10** in the MS source corresponding at $[C_{17}H_{18}O_6 - H_2O_2]^+$. Suggested pathway is proposed in Figure 1. NMR analyses accounted for structure **a**. Double-bond equivalent (DBE) value for this structure is 9. Analysis of ^{1}H NMR spectra and correlating its information with that of ^{13}C NMR spectrum resulted to the identification of two aromatic rings (cinnamoyl and furan moieties). The ^{13}C NMR spectrum (CD_3OD) showed 17 signals arising from two carbonyls, four sp^2 quaternary carbons, eight sp^2 methines, one methyl and one carboxylate group (SI4). In the ^{1}H NMR spectrum, characteristic protons of an α, β unsaturated ketone were observed at δ_H 6.77 (1H, d, *J* = 15.93) and 6.23 (1H, d, *J* = 15.93), typical signals of a cinnamoyl moiety, corroborated in COSY (Figure 2). Methine proton is observed at δ_H 3.80 (1H, s) in furan cycle. For the ester group, the methylene proton at δ_H 4.19, (2H, m) correlated in COSY with methyl proton at δ_H 1.27 (3H, t, *J* = 7.1). In addition, it correlated in HMBC with the quaternary carbons C-6′ at δ_C 171.9 and C-2′ at δ_C 151.3 (Figure 2). Location of the quaternary sp^2 carbon at C-5′ in furan cycle was evidenced by HMBC J^3 correlation of H-8 (cinnamoyl moiety) and J^2 correlation with H-4′. Connection between the furan and cinnamoyl moieties was also corroborated by J^3 correlation of H-4′ with carbonyl at C-9 (δ_C 200.8). Location of carbonyl at C-6′ in ester group was evidenced by the correlation in HMBC J^3 with H7′ and correlations in J^4 of H-4′, H-9′. The methyl at δ_H 1.97 (3H, s) and the carboxylate substitutions were observed in *meta* and *ortho* of the oxygen bridge in the furan cycle respectively.

Figure S5-1. Suggested MS source decomposition pathway for compound **10**.

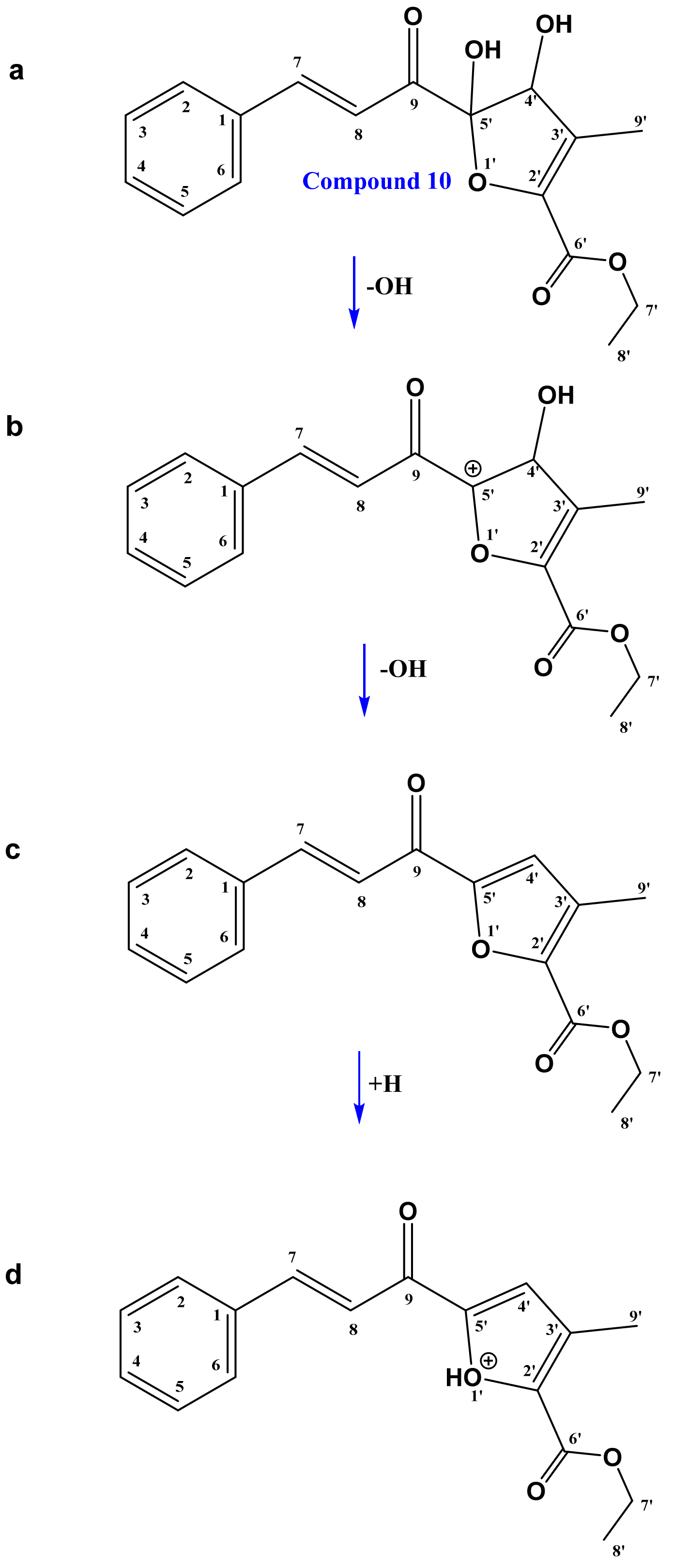

Figure S5-2: Main HMBC and COSY correlations for compound **10**.

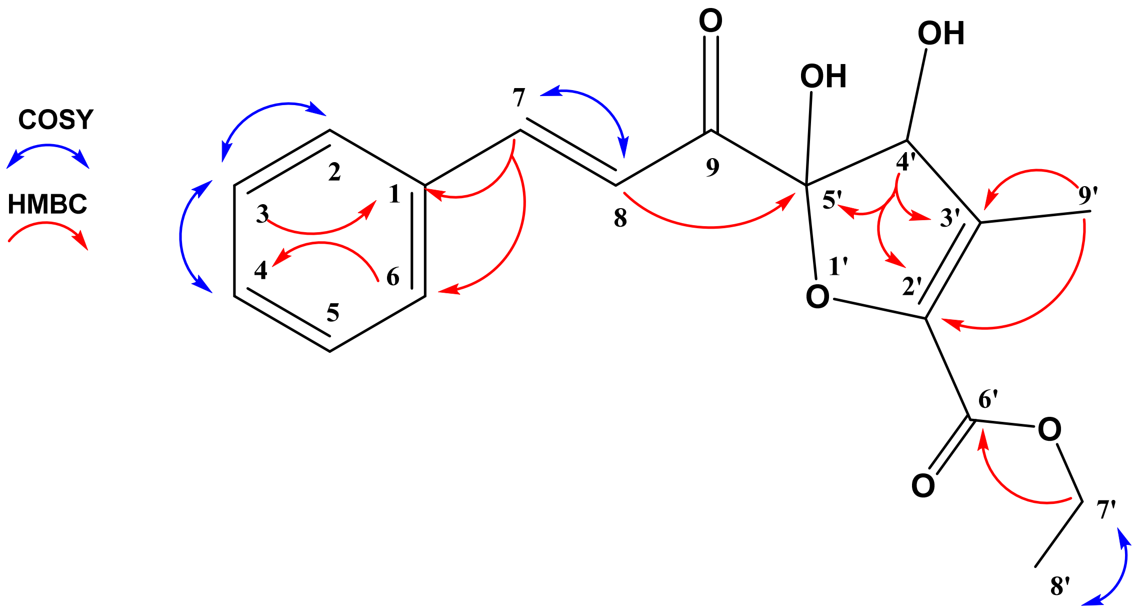

SI 6. CPC fractograms of cyclohexane extract (PCC) from *P. coruscans*

Figure S6. CPC fractograms of cyclohexane extract (PCC) from *P. coruscans*

Total CPC UV fractogram at 280 nm. Fraction volume is 0,5 ml.

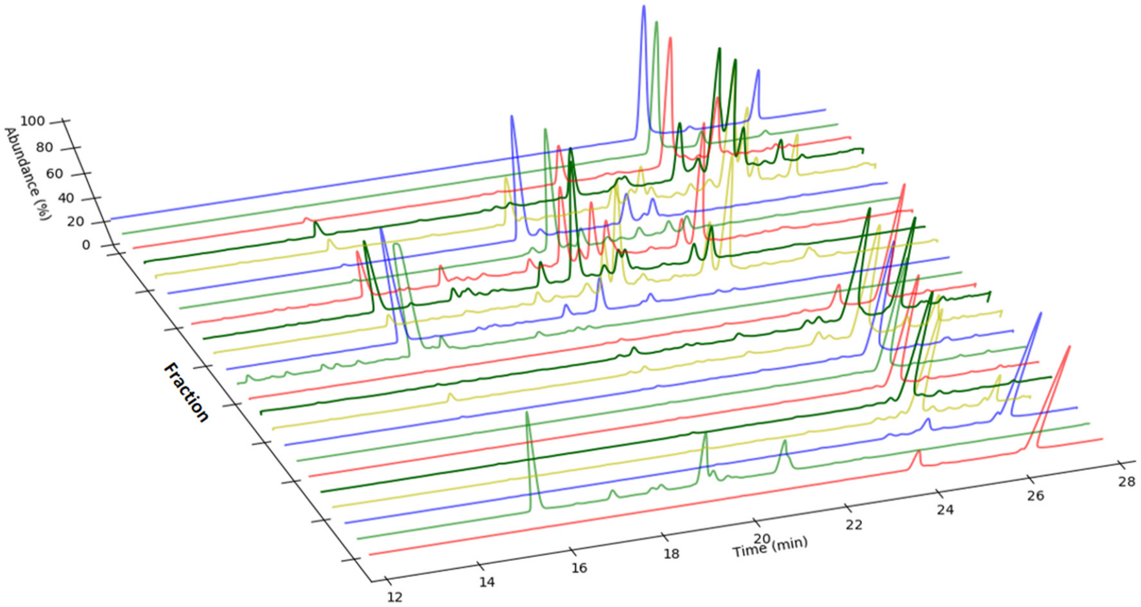

CPC fractogram at *m/z* 285 (mass of compound **4**). Fraction volume is 0,5 ml.

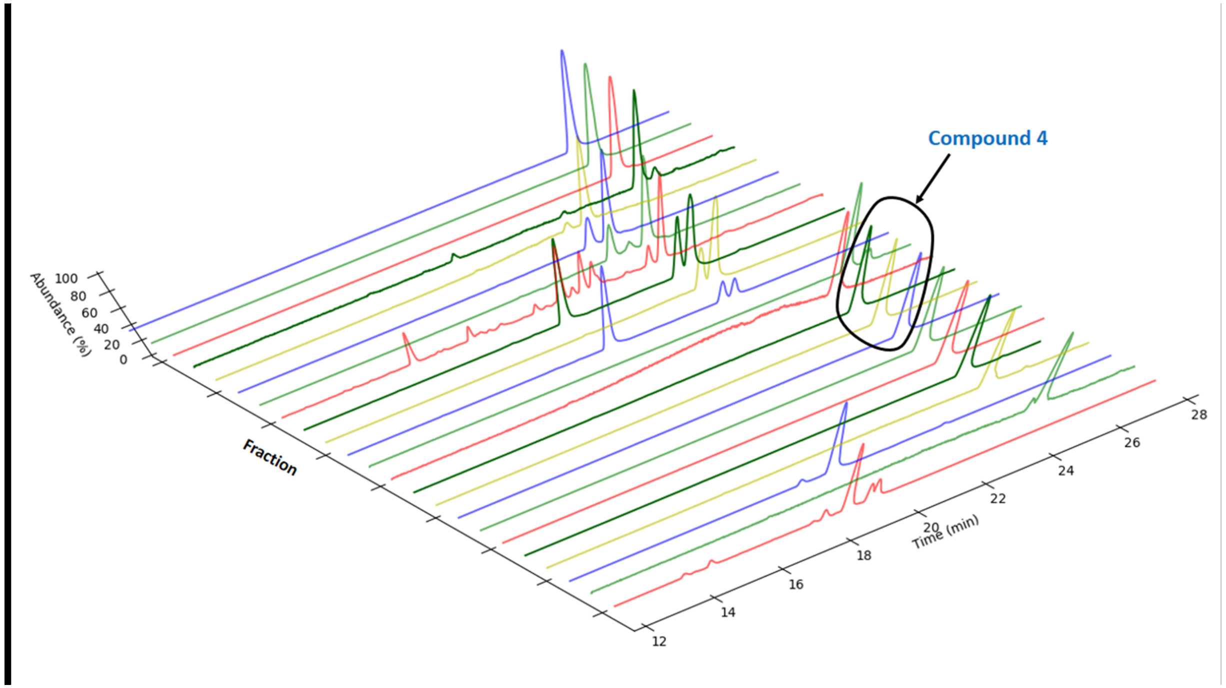

CPC fractogram at *m*/*z* 299 (mass of compounds **1** and **5**). Fraction volume is 0,5 ml.

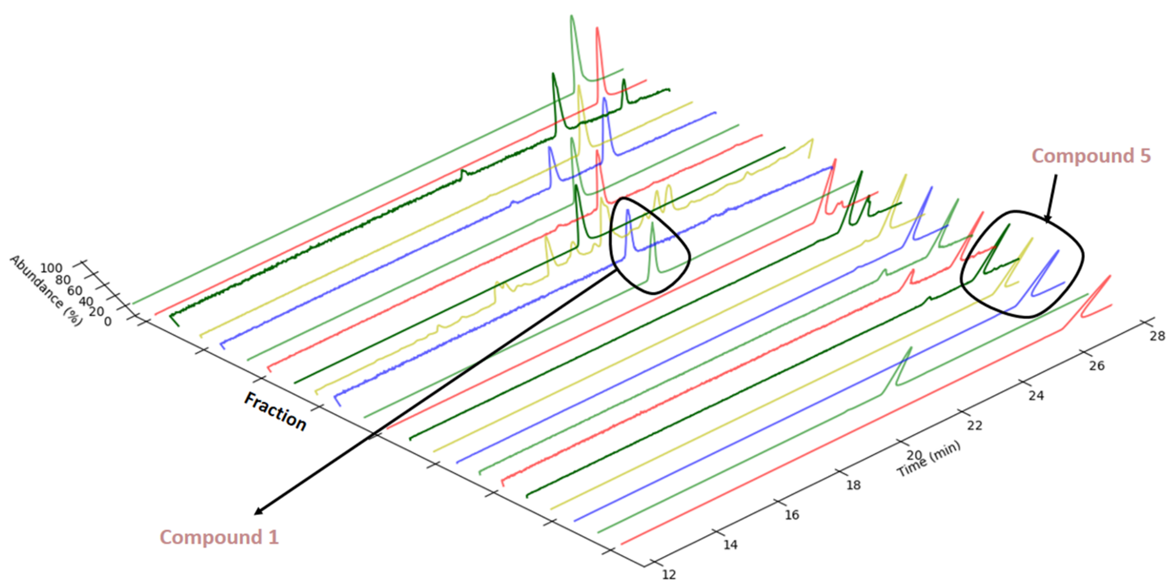

SI 7. Proton NMR of known compounds isolated for *P. coruscans*

Figure S7. Proton NMR of known compounds isolated for *P. coruscans*

^{1}H NMR for compound **1** in CD_3CN

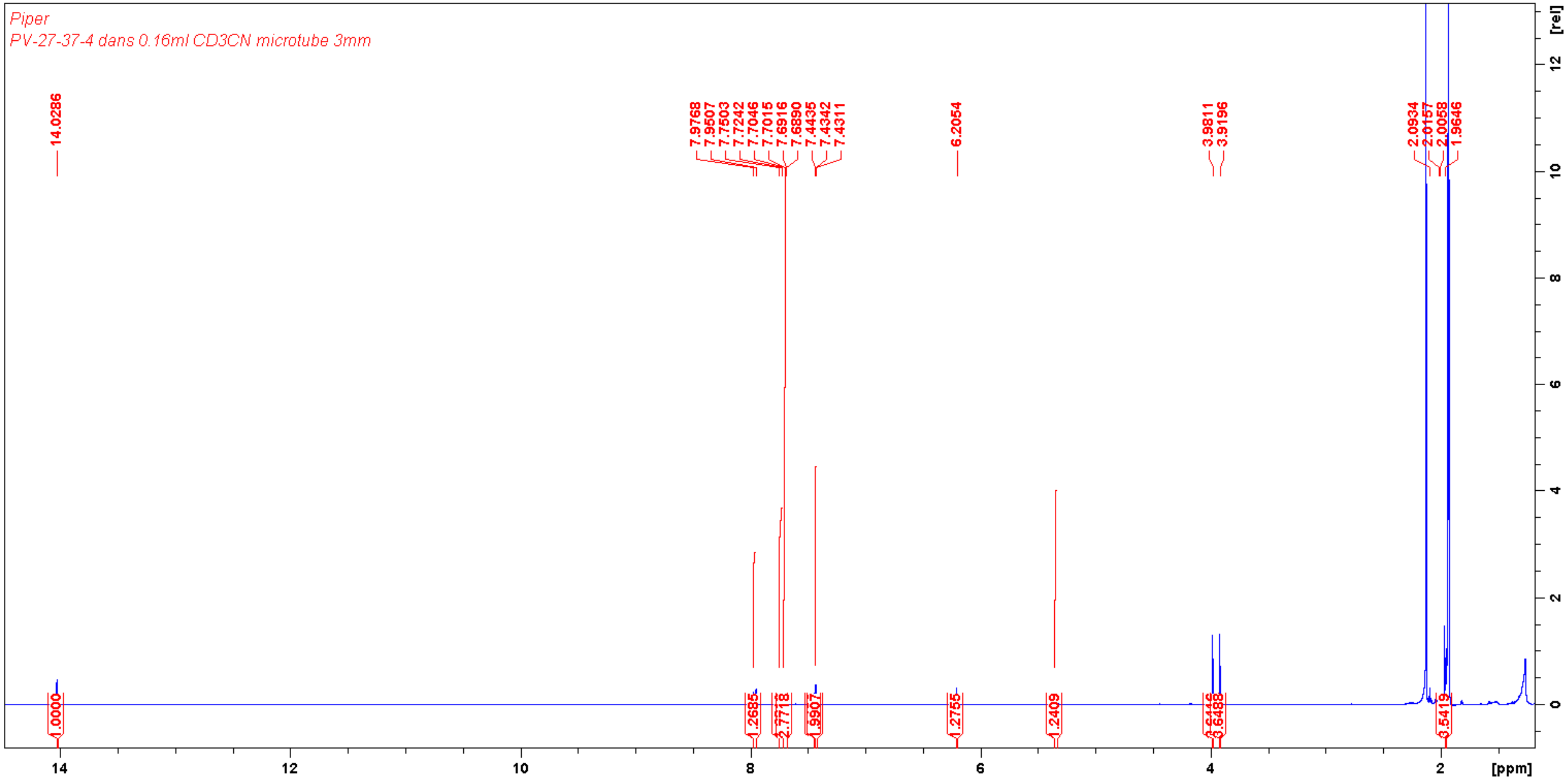

^{1}H NMR for compound **2** in $CDCl_3$

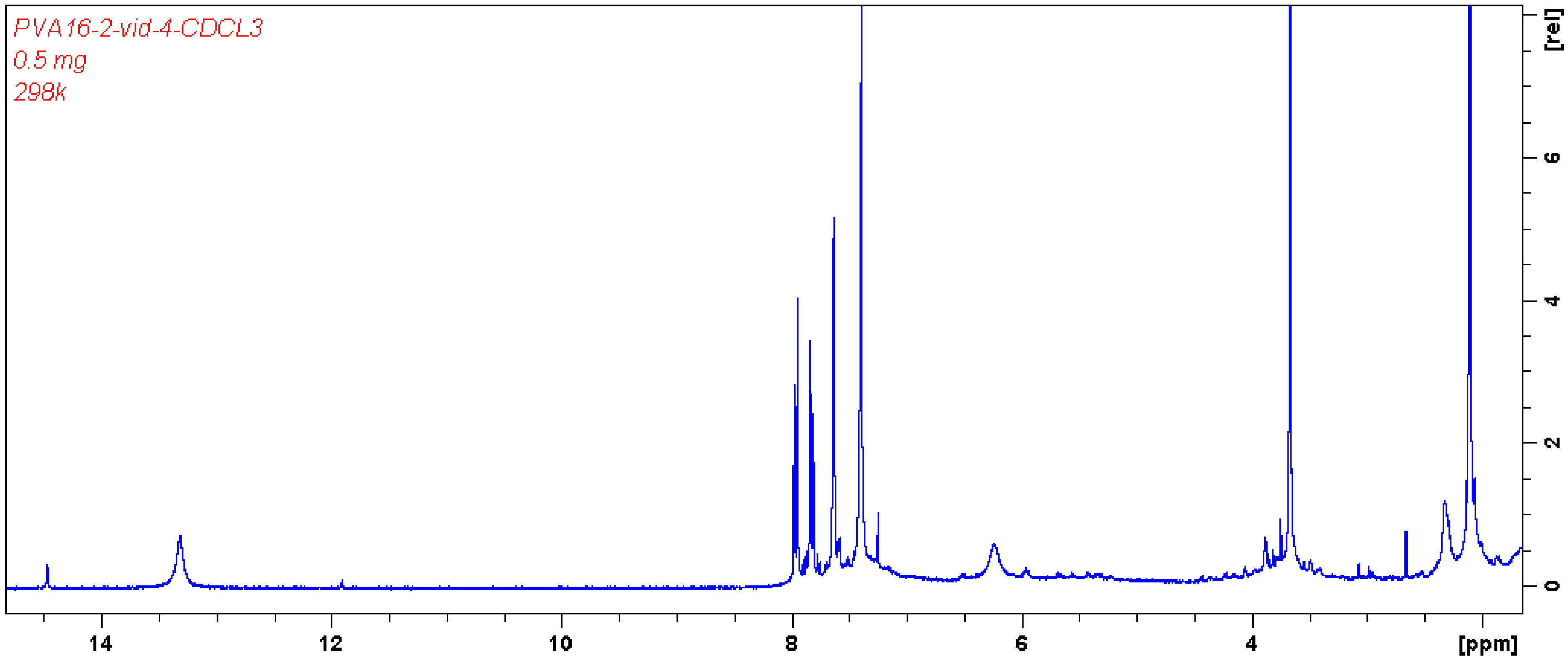

^{1}H NMR for compound **3** in $CDCl_3$

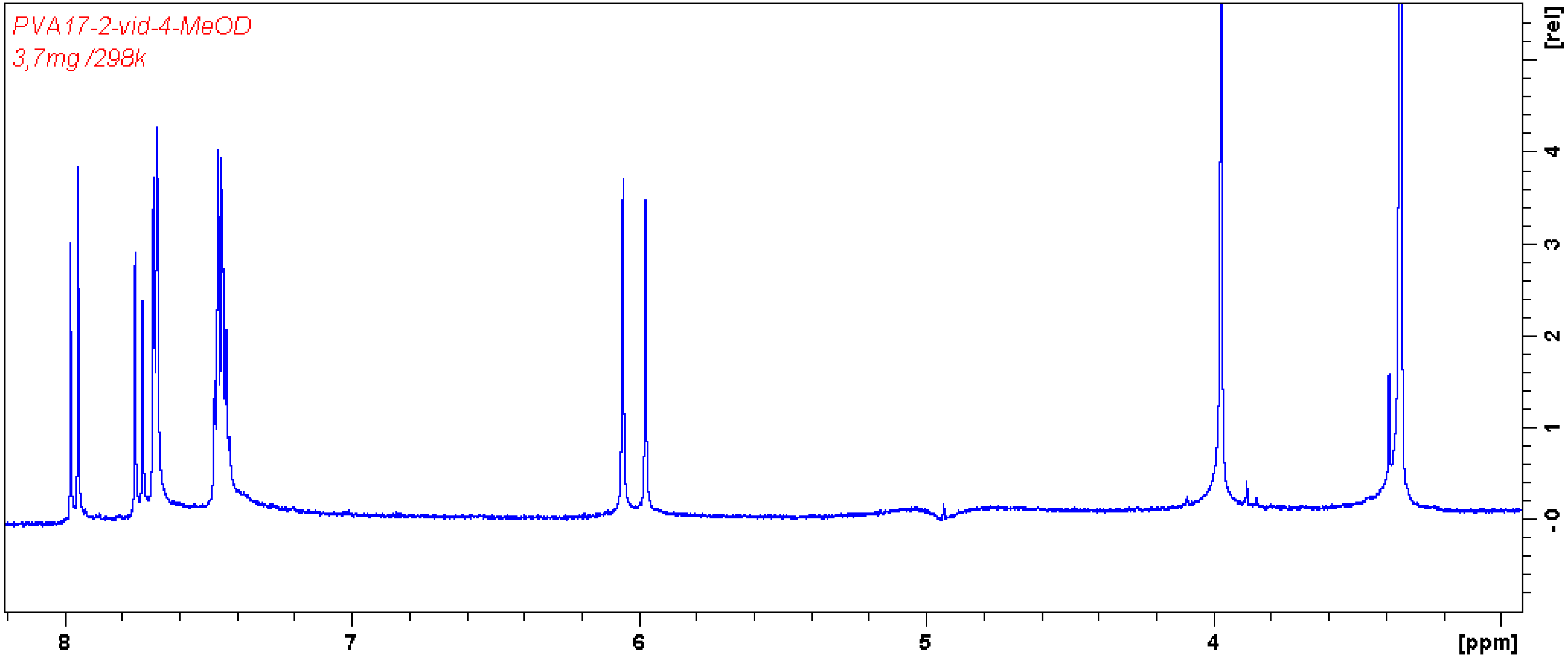

^{1}H NMR for compound **4** in CD_3CN

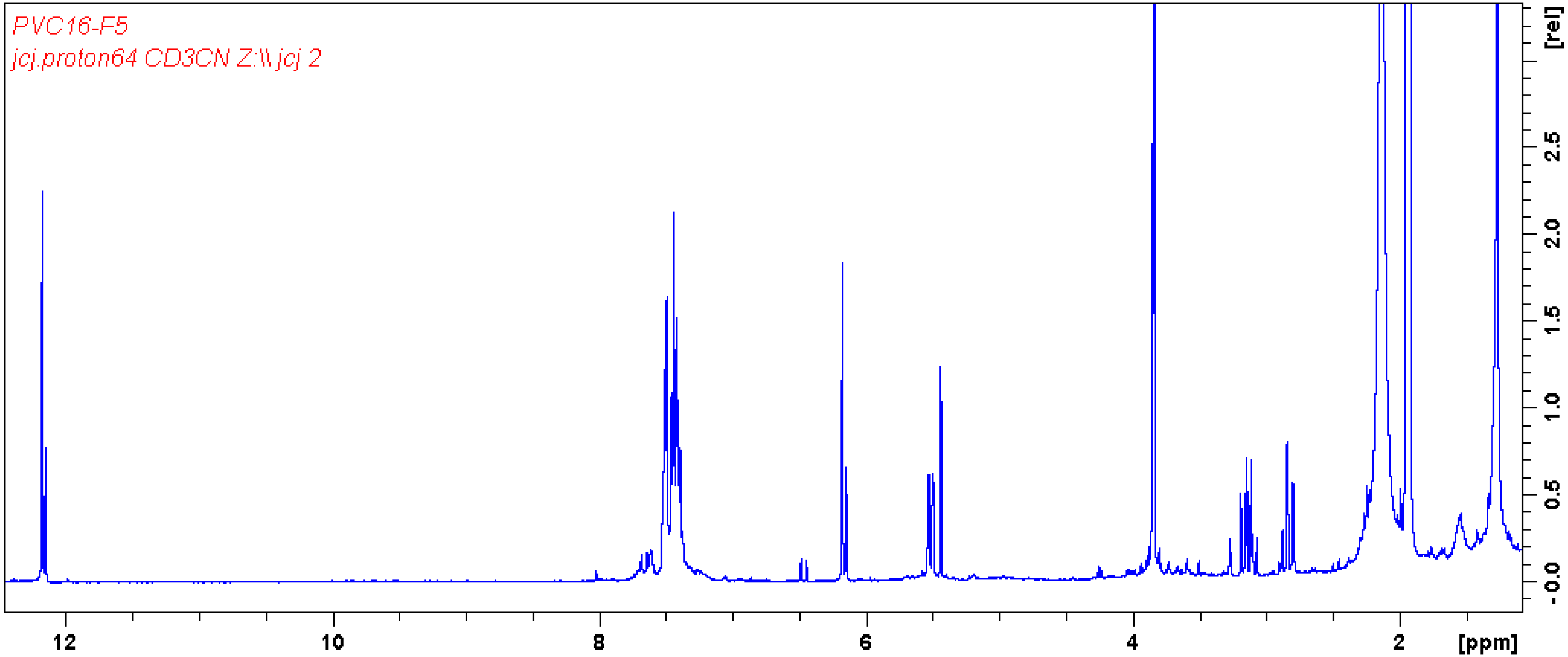

^{1}H NMR for compound **5** in CD_3CN

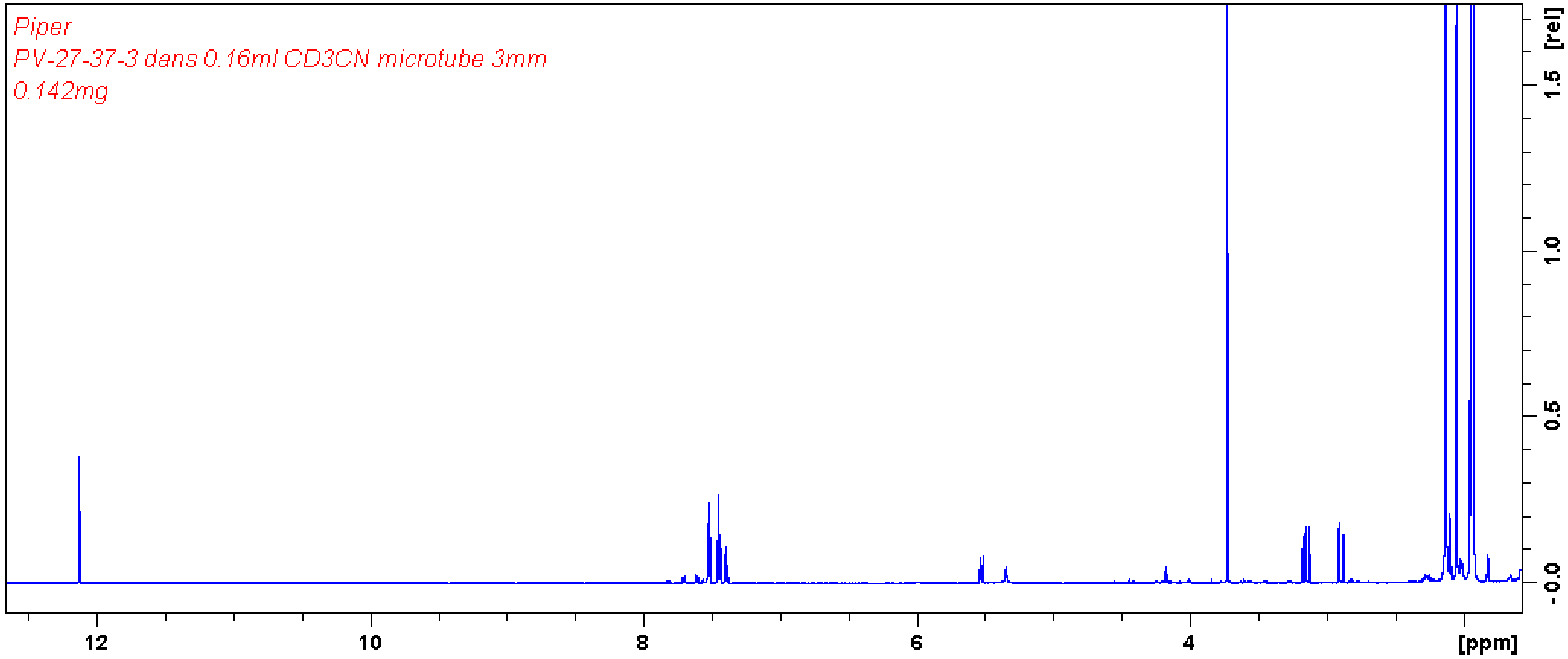

^{1}H NMR for compound **6** in $CDCl_3$

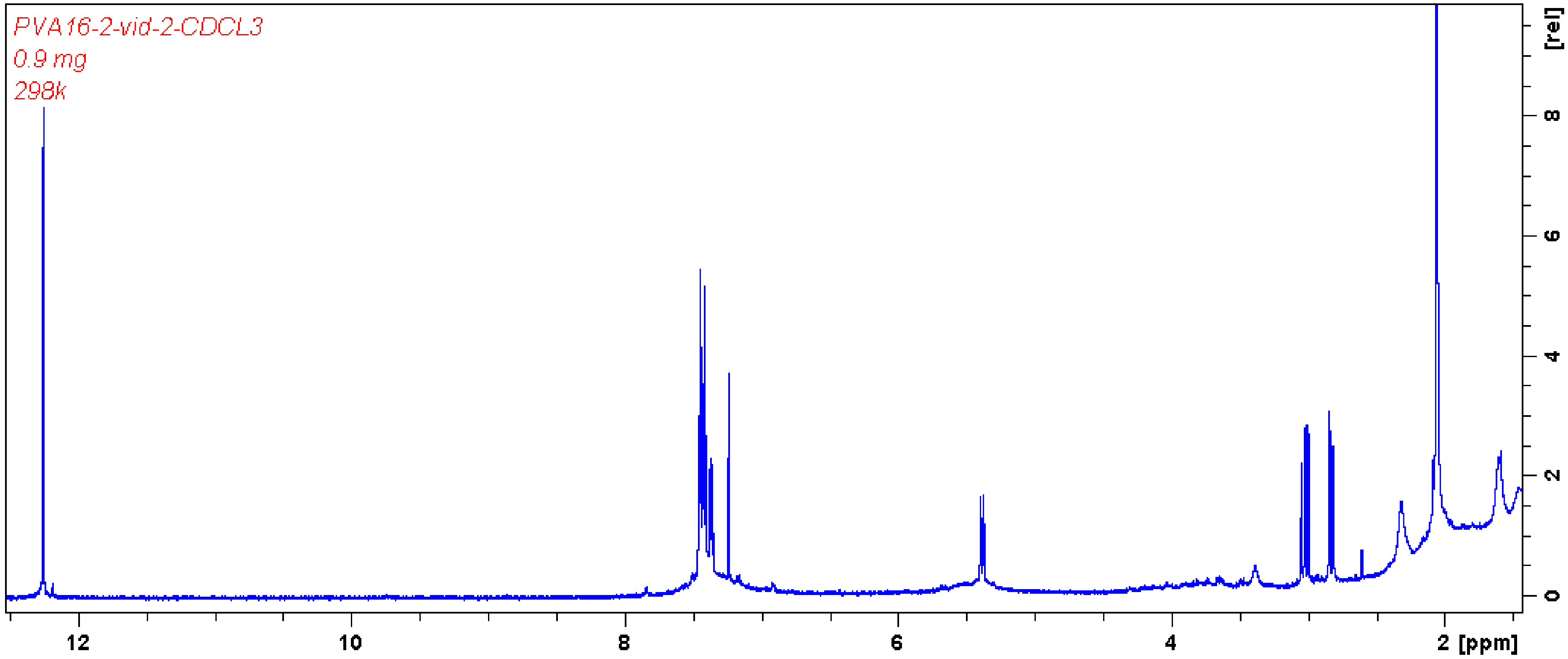

^{1}H NMR for compound **7** in CD_3OD

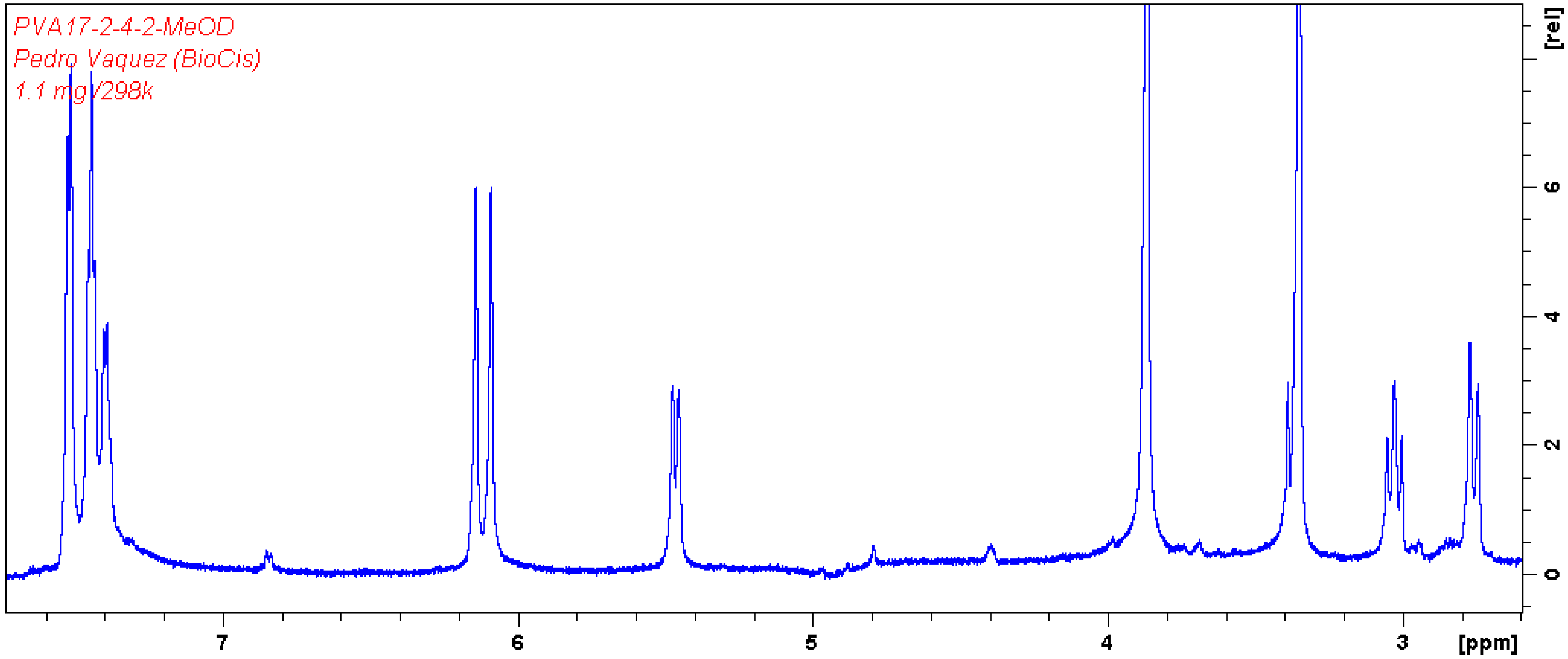

^{1}H NMR for compound **8** in CD_3OD

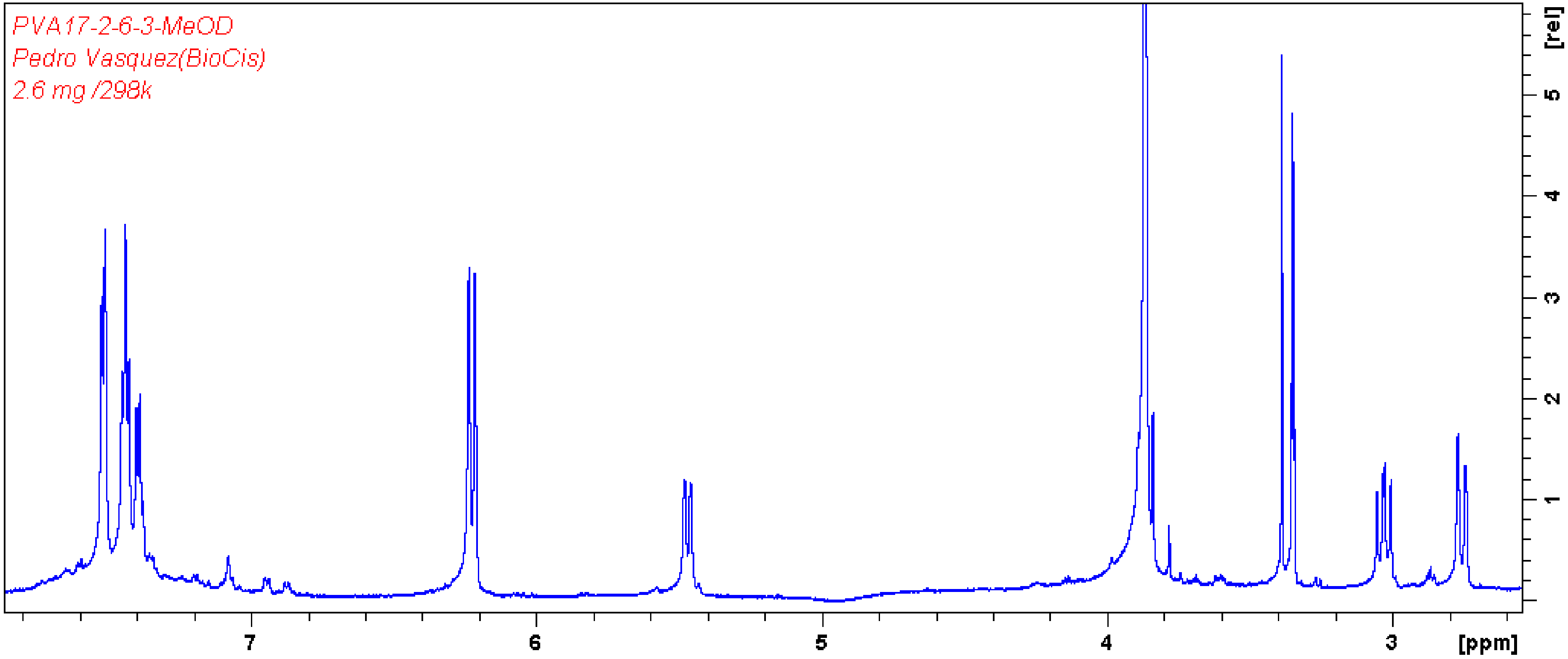

^{1}H NMR for compound **9** in CD_3OD

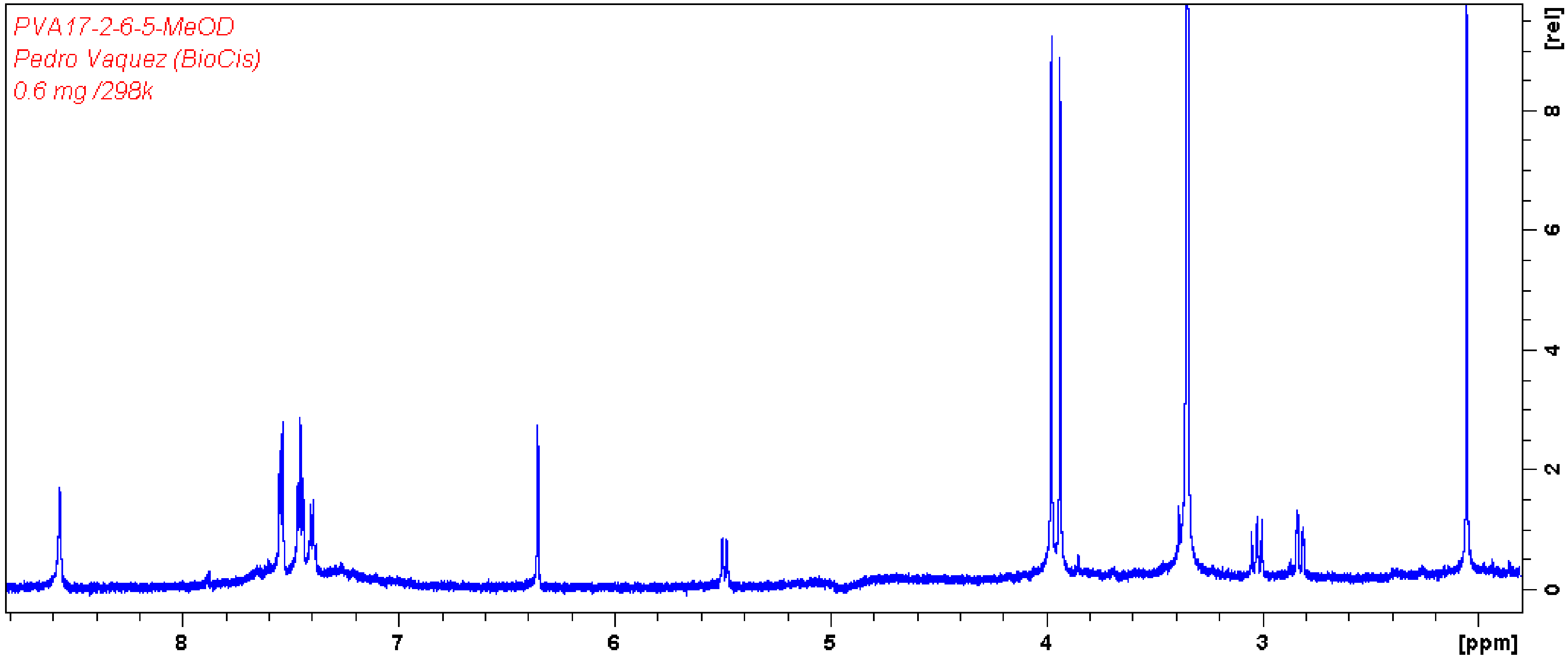

^{1}H NMR for compound **11** in CD_3OD

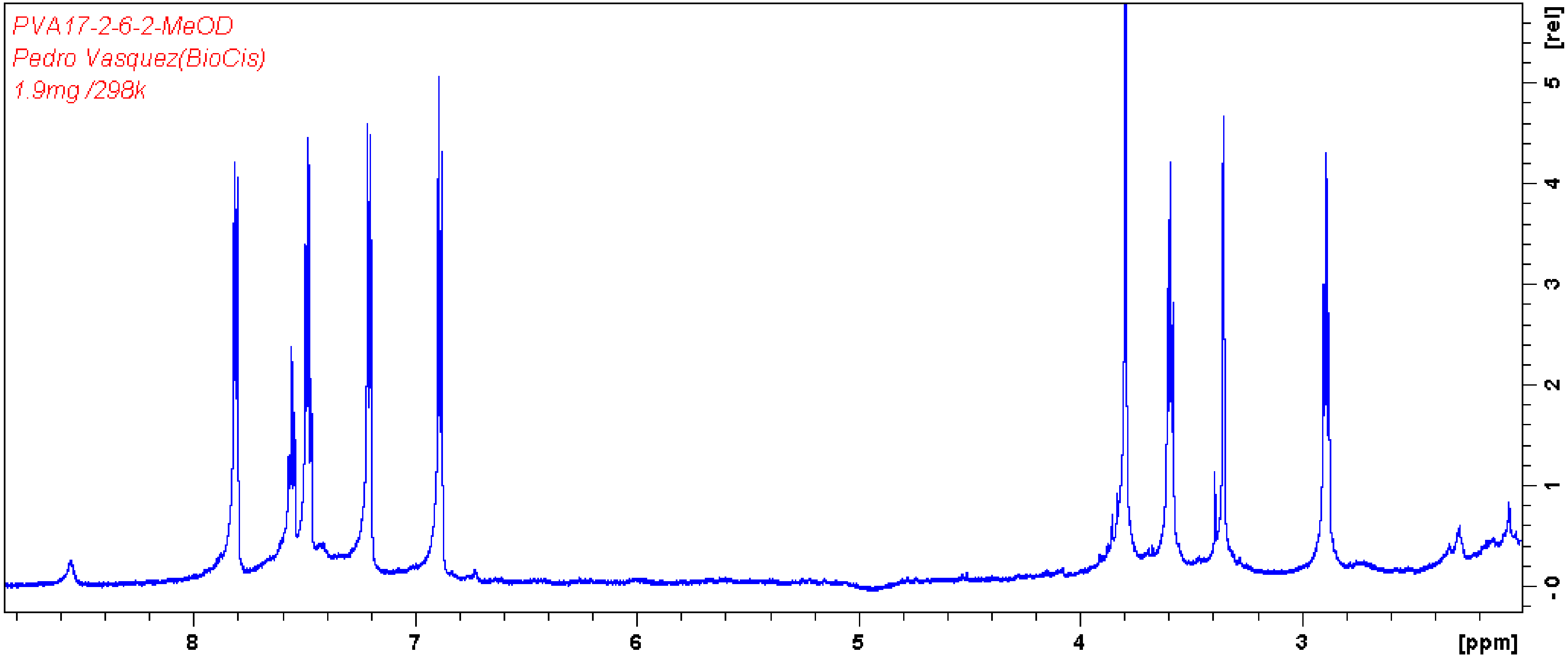

^{1}H NMR for compound **12** in CD_3OD

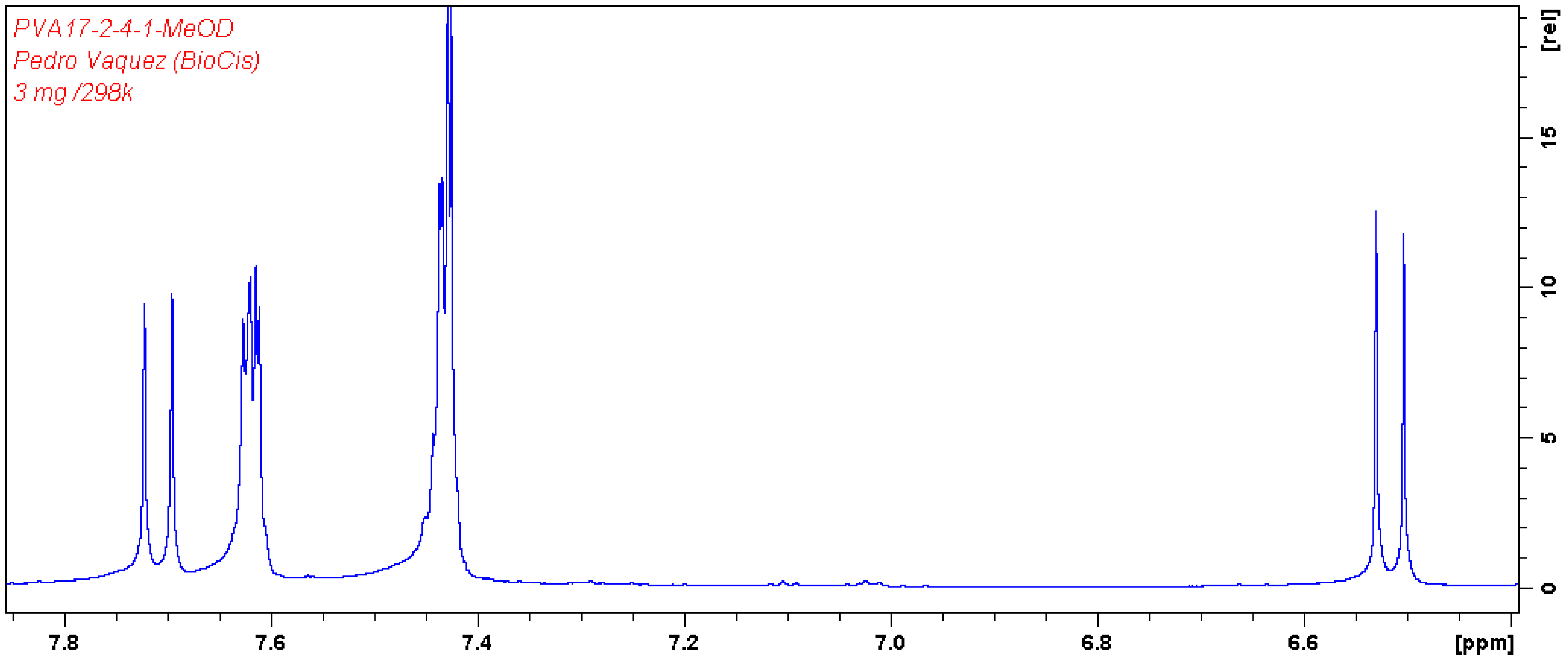

SI 8. 1D and 2D NMR for compound **10**

Figure S8. 1D and 2D NMR for compound **10**

^{1}H NMR for compound **10** in CD_3OD

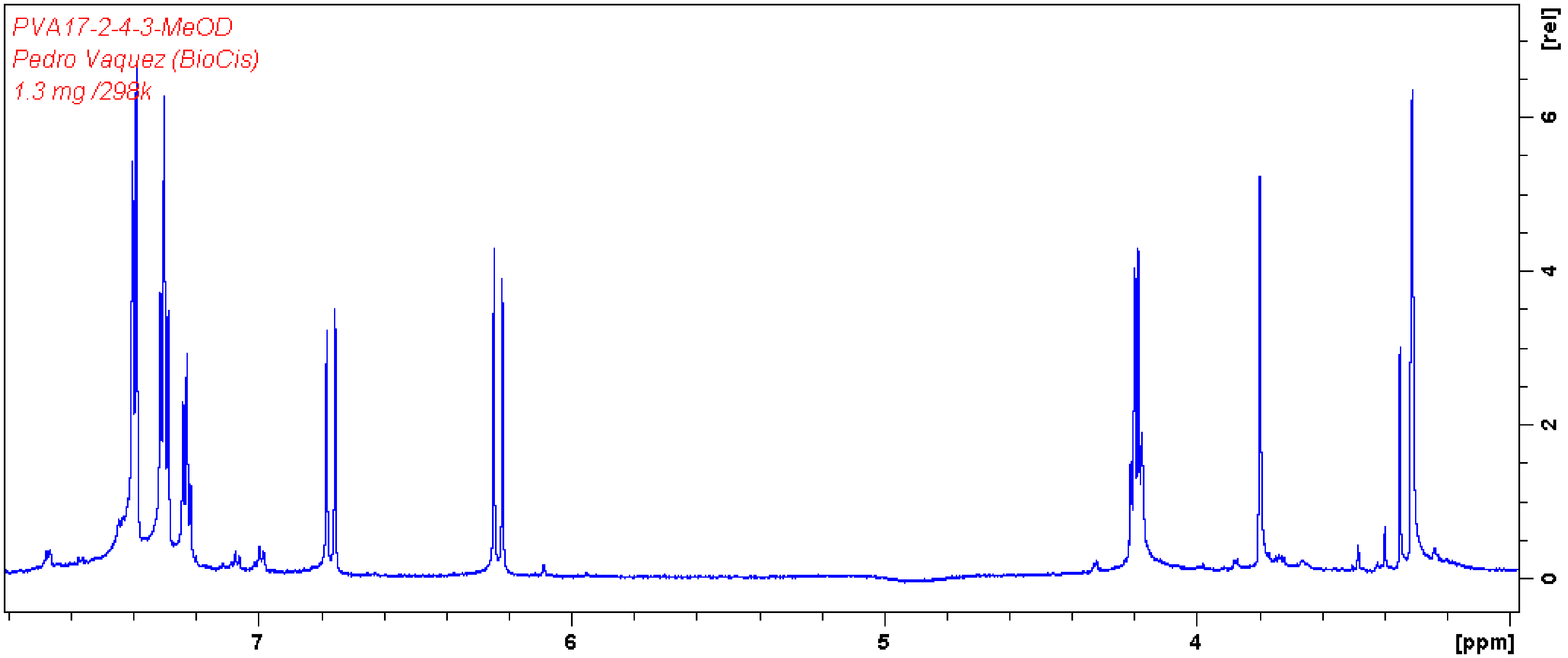

COSY spectrum for compound **10**

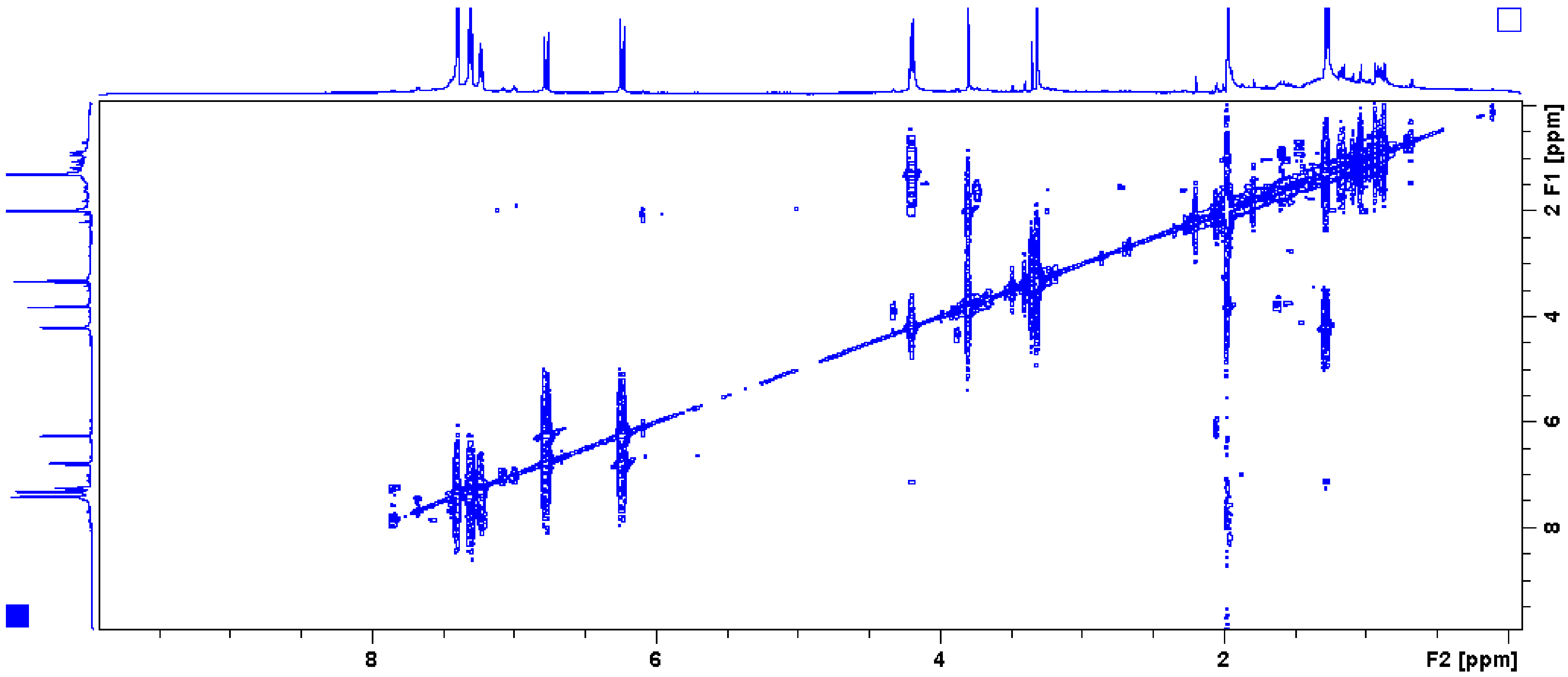

HSQC spectrum for compound **10**

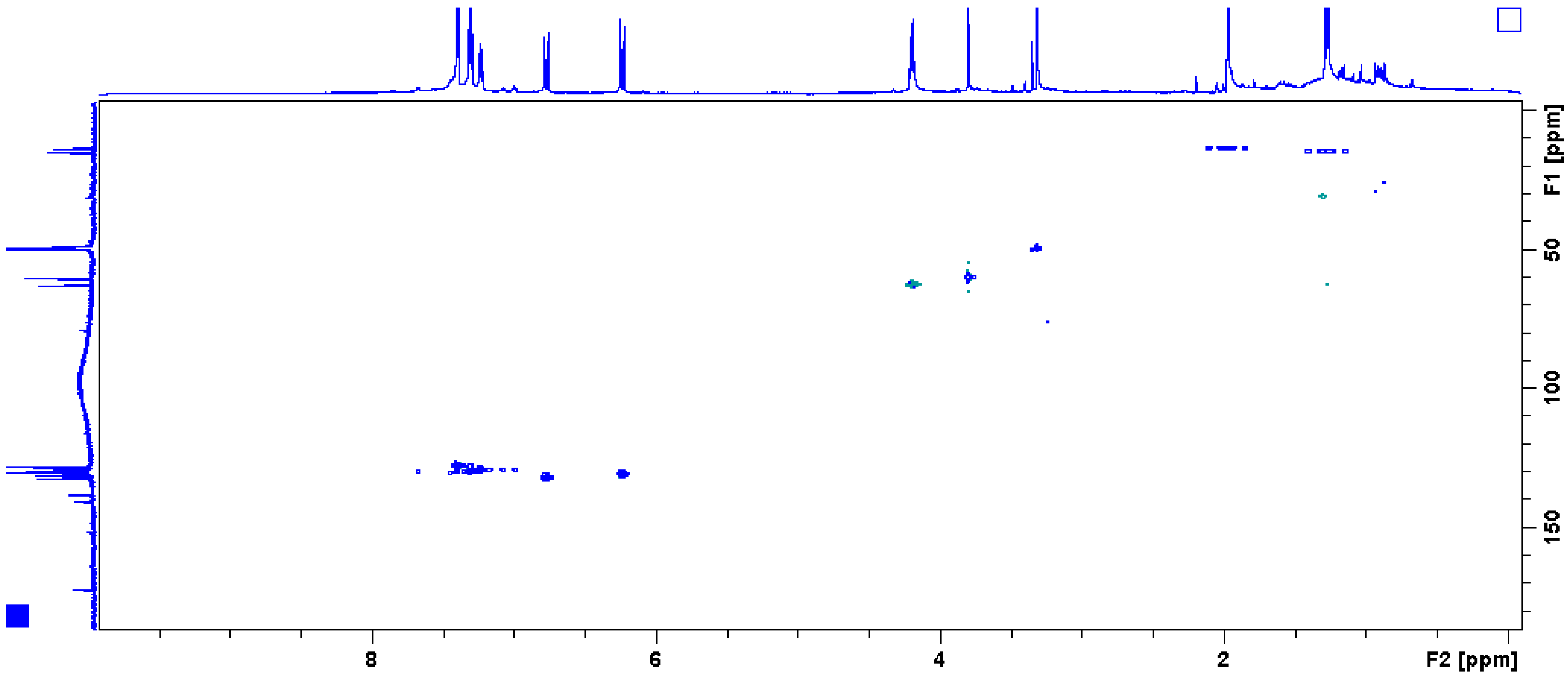

HMBC spectrum for compound **10**

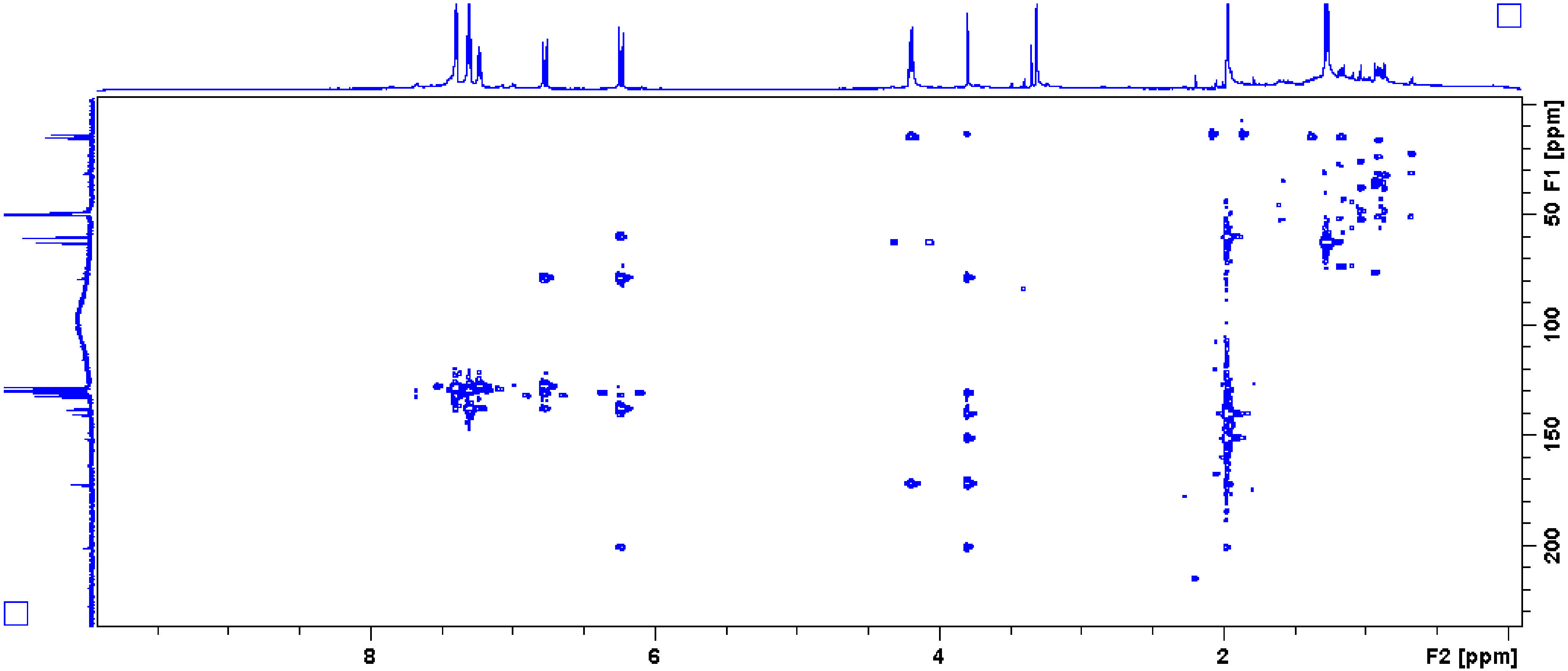

^{13}C NMR spectrum for compound **10**

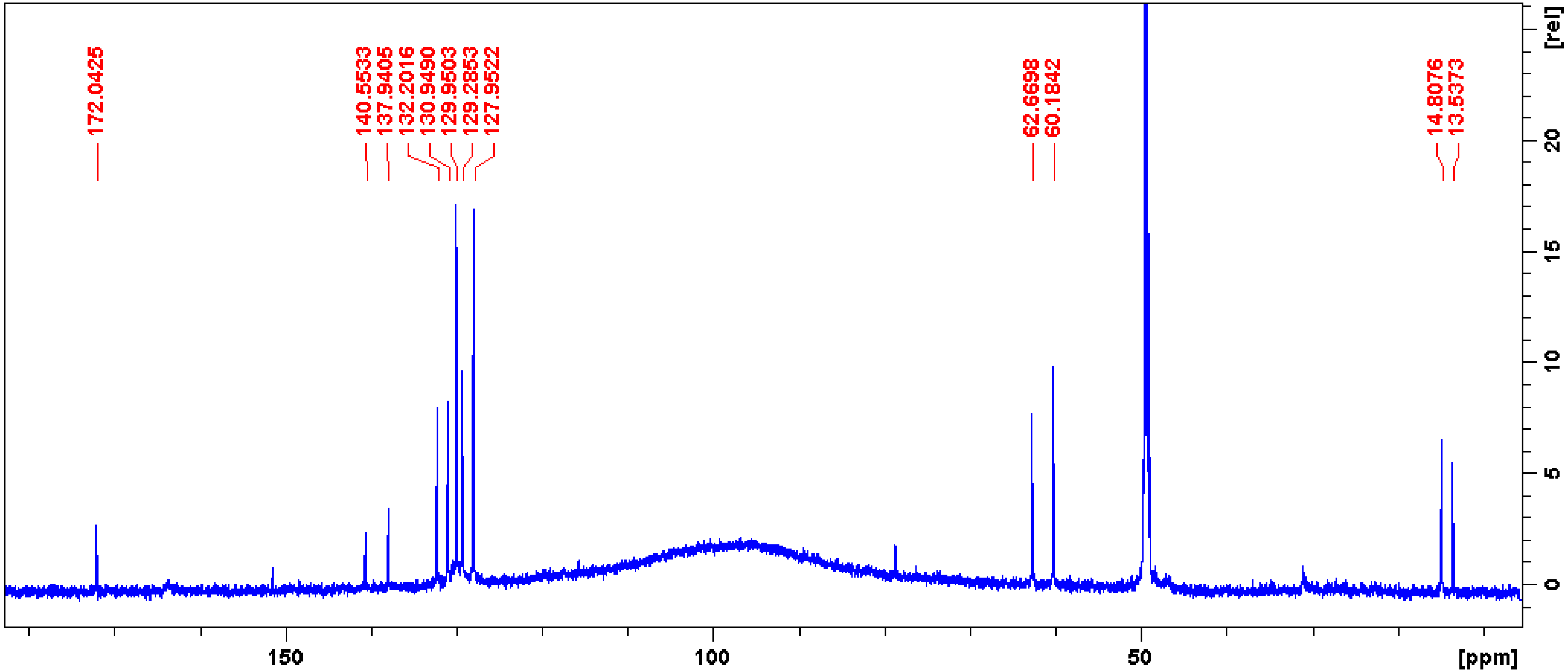

SI 9. NMR spectroscopic data for isolated compounds

Table S1. NMR spectroscopic data for isolated compounds

NMR spectroscopic data for flavanones **4-9**.

**Position**	**4**		**5**		**6**		**7**		**8**		**9**	
	δ_H, mult. (*J* in Hz)	δ_C	δ_H, mult. (*J* in Hz)	δ_C	δ_H, mult. (*J* in Hz)	δ_C	δ_H, mult. (*J* in Hz)	δ_C	δ_H, mult. (*J* in Hz)	δ_C	δ_H, mult. (*J* in Hz)	δ_C
**1**												
**2**	5.51, dd (*2.8, 12.7*)	80.2	5.52, dd (*3.1, 12.7*)	79.6	5.39, dd (2.9, *12.9*)	78.9	5.46, dd (*2.4, 12.6*)	80.5	5.47, dd (*1.9, 13.0*)	80.4	5.49, dd (2.3, *12.6*)	80.2
**3**	a)3.14, dd (*12.7, 17.2*)<br>b)2.83, dd (*3.2, 17.1*)	43.6	a) 3.15, dd (*12.7, 17.1*)<br>b) 2.89, dd (*3.1, 17.1*)	43.7	b) 3.02, dd (*13.0, 17.1*)<br>a) 2.83, dd (*3.1, 17.0*)	43.7	a) 3.02, dd (*13.0, 16.4*)<br>b) 2.76, dd (*2.3, 16.3*)	46.6	a) 3.03, dd (*13.0, 16.6*)<br>b) 2.76, dd (*2.2, 16.6*)	46.5	a) 3.03, dd (*12.7, 16.9,*)<br>b) 2.83, dd (*2.5, 16.3*)	46.8
**4**		197.7		199.1		196.6		191.9		191.9		192.9
**4a**		103.7		105.9		103.1		106.1		106.7		106.4
**5**		161.2		160.0		159.4		164.6		163.9		166.0
**6**		106.2		111.8		102.4	6.14, s	94.6	6.23, d (*1.8*)	94.0	6.36, s	90.0
**7**		166.8		166.2		161.1		167.2		168.4		162.4
**8**	6.17, s	91.9		110.5		103.2	6.09, s	97.4	6.24, d (*1.8*)	95.1		107.2
**8a**		162.5		159.0		157.8		166.5		166.8		159.5
**1′**		140.0		140.1		139.0		140.9		140.5		141.1
**2′**	7.51, d (*6.8*)	127.4	7.52, d (*7.4*)	127.3	7.45, d (*7.1*)	126.1	7.52, d (*7.2*)	127.4	7.51, d (*7.3*)	127.4	7.55, d (*7.9*)	127.3
**3′**	7.44, t (*7.2*)	129.7	7.44, t (*7.5*)	129.7	7.42, dd (*7.5*)	129.0	7.44, t (*7.0*)	129.8	7.44, t (*7.4*)	129.8	7.46, t (*7.5*)	130.0
**4′**	7.39, t (*7.1*)	129.7	7.39, t (*7.3*)	129.6	7.36, dd (*7.1*)	128.6	7.40, t (*7.3*)	129.6	7.39, t (*7.1*)	129.7	7.40, t (*7.1*)	129.7
**5′**	7.44, t (*7.2*)	129.7	7.44, t (7.5)	129.7	7.42, dd (*7.5*)	129.0	7.44, t (*7.0*)	129.8	7.44, t (*7.4*)	129.8	7.46, t (*7.5*)	130.0
**6′**	7.51, d (*6.7*)	127.4	7.52, d (*7.4*)	127.3	7.45, d (*7.1*)	126.1	7.52, d (*7.2*)	127.4	7.51, d (*7.3*)	127.4	7.55, d (*7.9*)	127.3
**O-CH3**	3.84, s	7) 56.9	7) 3.72, s	60.8			5) 3.86, s	56.4	5) 3.869, s<br>7) 3.867, s	56.5<br>56.5	5) 3.98, s<br>7) 3.94, s	-56.5<br>-56.4
**OH**	12.17, s		5) 12.12		5) 12.25, s							
**CH_3**	1.94, s	6) 7.1	6) 2.04, s<br>8) 2.04, s	8.6<br>8.1	6) 2.060, s<br>8) 2.055, s	6) 7.2<br>8) 7.7					2.06, s	8)8.2

NMR spectroscopic data for chalcones **1-3**.

**Position**	**1**		**2**		**3**	
	δH, mult. (*J* in Hz)[a]	δC[a]	δH, mult. (*J* in Hz)[b]	δC[b]	δH, mult. (*J* in Hz)[c]	δC[c]
**1**		135.4		135.3		137.3
**2**	7.70, dd (*1.9, 7.8*)	128.4	7.63, d (*6.5)*	128.5	7.68, d (*6.3)*	129.6
**3**	7.43, m	130.2	7.40 m	129.0	7.45, m	131.5
**4**	7.44, m	129.0	7.40 m	130.3	7.47, m	130.3
**5**	7.43, m	130.2	7.40 m	129.0	7.45, m	131.5
**6**	7.70, m	128.4	7.63, d (*6.5)*	128.5	7.68, d (*6.3)*	129.6
**α**	7.97, d (*15.6*)	128.0	7.95, d (*15.5)*	126.4	7.96, d (*15.6)*	129.3
**β**	7.74, d (*15.6*)	141.6	7.82, d (*15.5)*	143.2	7.74, d (*15.6)*	143.2
**9**		193.1		194.2		193.1
**1′**		105.8		109.7		107.0
**2′**		163.8		162.5		169.1
**3′**		104.9		110.2	5.98, d (*2.2)*	97.4
**4′**		164.1		160.7		167.1
**5′**	6.21, s	87.2	6.23, s	99.8	6.06, d (*2.0)*	92.8
**6′**		161.6		161.4		165.1
**O-CH3**	4) 3.92, s<br>6) 3.99, s	-55.6<br>-55.9	6′)3.66, s	62.3	6′) 3.97, s	56.7
**OH**	2′) 14.03	163.8	2′) OH<br>4′) OH	162.5<br>160.7	2′) OH<br>4′) OH	169.1<br>167.1
**CH_3**	3′) 1.96, s	6.5	2.1, s	8.1	-	

NMR spectroscopic data for compound **12**

**Position**	**12**	
	δH, mult. (*J* in Hz)[a]	δC[a]
**1**		170.7
**2**	6.52, d (*16.0*)	119.6
**3**	7.71, d (*16.0*)	146.6
**4**		136.1
**5**	7.62, dd (*7.6, 4.1*)	129.5
**6**	7.43, dd (*4.8, 1.6*)	130.3
**7**	7.62, dd (*7.6, 4.1*)	129.5
**8**	7.43, dd (*5.18*)	131.7
**9**		136.1

NMR spectroscopic data for compound **10**

**Position**	**10**	
	δ_H, mult. (*J* in Hz)	δ_C
**1**		137.8
**2**	7.40, d (*7.7*)	127.9
**3**	7.30, dd (*7.7, 7.4*)	129.9
**4**	7.23, t (*7.4, 7.1*)	129.2
**5**	7.30, dd (*7.7, 7.4*)	129.9
**6**	7.40, dd (*7.7*)	127.9
**7**	6.77, d (*15.9*)	132.1
**8**	6.23, d (*15.9*)	130.9
**9**		200.8
**2′**		151.3
**3′**		140.4
**4′**	3.80, s	60.0
**5′**		78.6
**6′**		171.9
**7′**	4.19, m	62.5
**8′**	1.27, t (*7.1*)	14.9
**9′**	1.97; s	13.5

NMR spectroscopic data for compound **11**

**Position**	**11**	
	δ_H, mult. (*J* in Hz)	δ_C
**1**		170.5
**2**		136.1
**3**	7.81, d (*7.2*)	128.5
**4**	7.48, t (*7.6*)	129.8
**5**	7.55, dd (7.3, *7.4*)	132.9
**6**	7.48, dd (*7.6*)	129.8
**7**	7.81, d (*7.2*)	128.5
**8**	NH	
**9**	3.59, t (*7.4*)	43.2
**10**	2.89, t (*7.3*)	36.0
**11**		132.9
**12**	7.21, d (*8.5*)	131.1
**13**	6.88, d (*8.6*)	115.2
**14**		160.0
**15**	6.88, d (*8.6*)	115.2
**16**	7.21, d (*8.5*)	131.1
**O-CH$_3$**	14) 3.79, s	55.8

SI 10. LCMS of compounds isolated from *P. coruscans*.

Figure S9. LCMS of compounds isolated from *P. coruscans*.

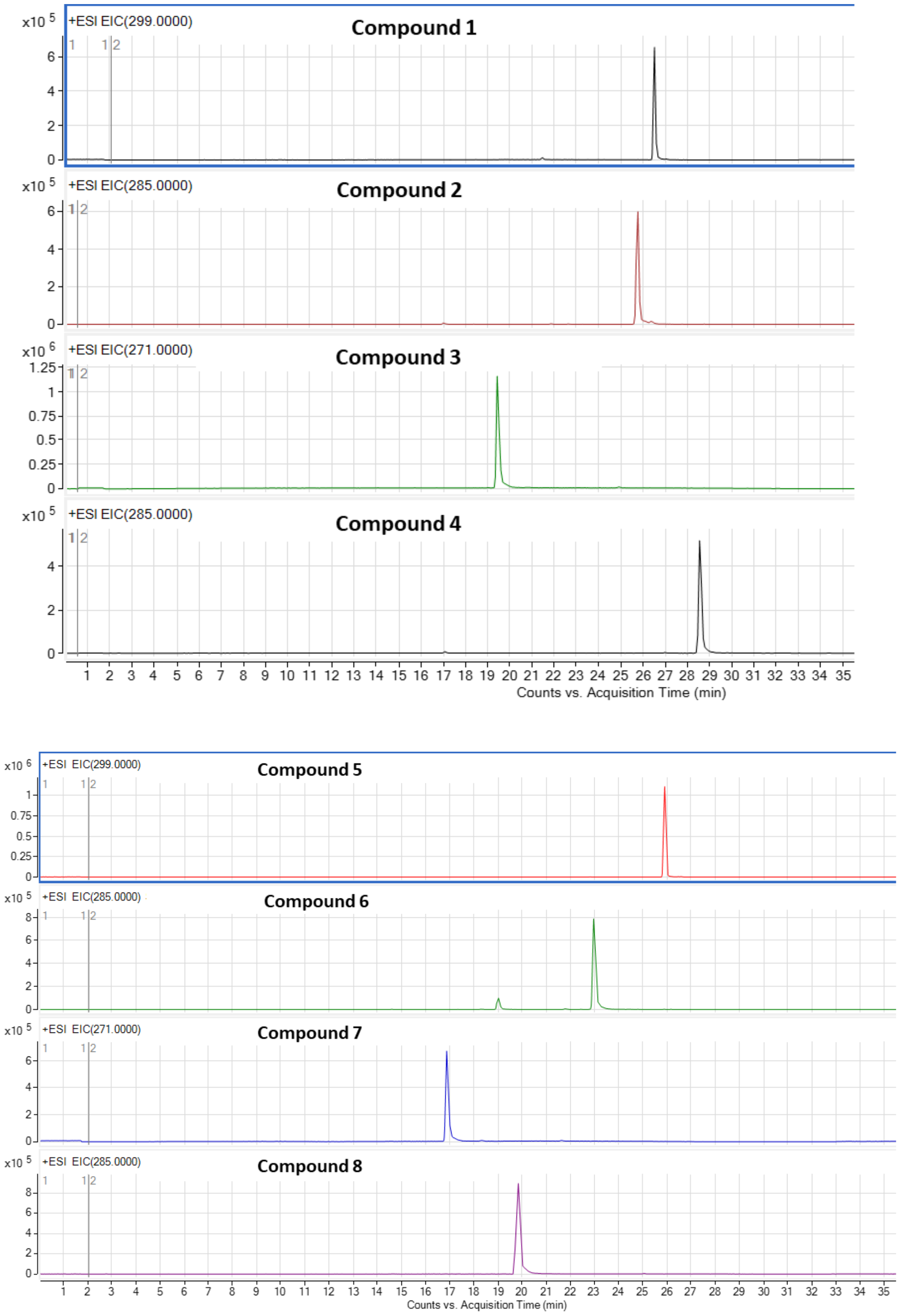

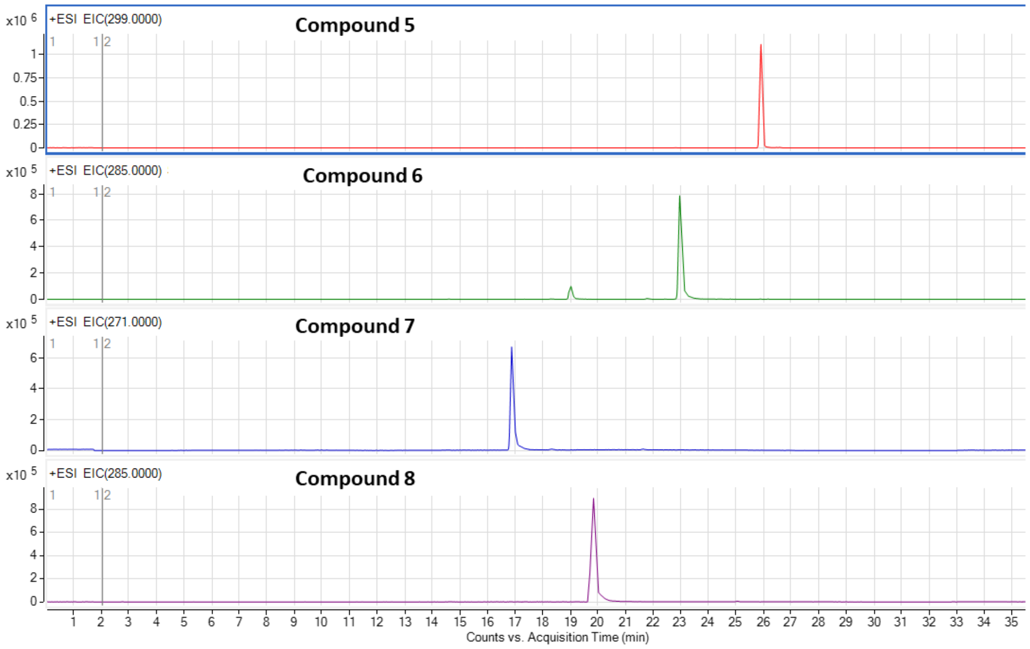

Compound 5
Compound 6
Compound 7
Compound 8
+ESI EIC(299.0000)
+ESI EIC(285.0000)
+ESI EIC(271.0000)
+ESI EIC(285.0000)
Counts vs. Acquisition Time (min)

SI 11. Structures of antimalarial drugs

Figure S10. Structures of antimalarial drugs

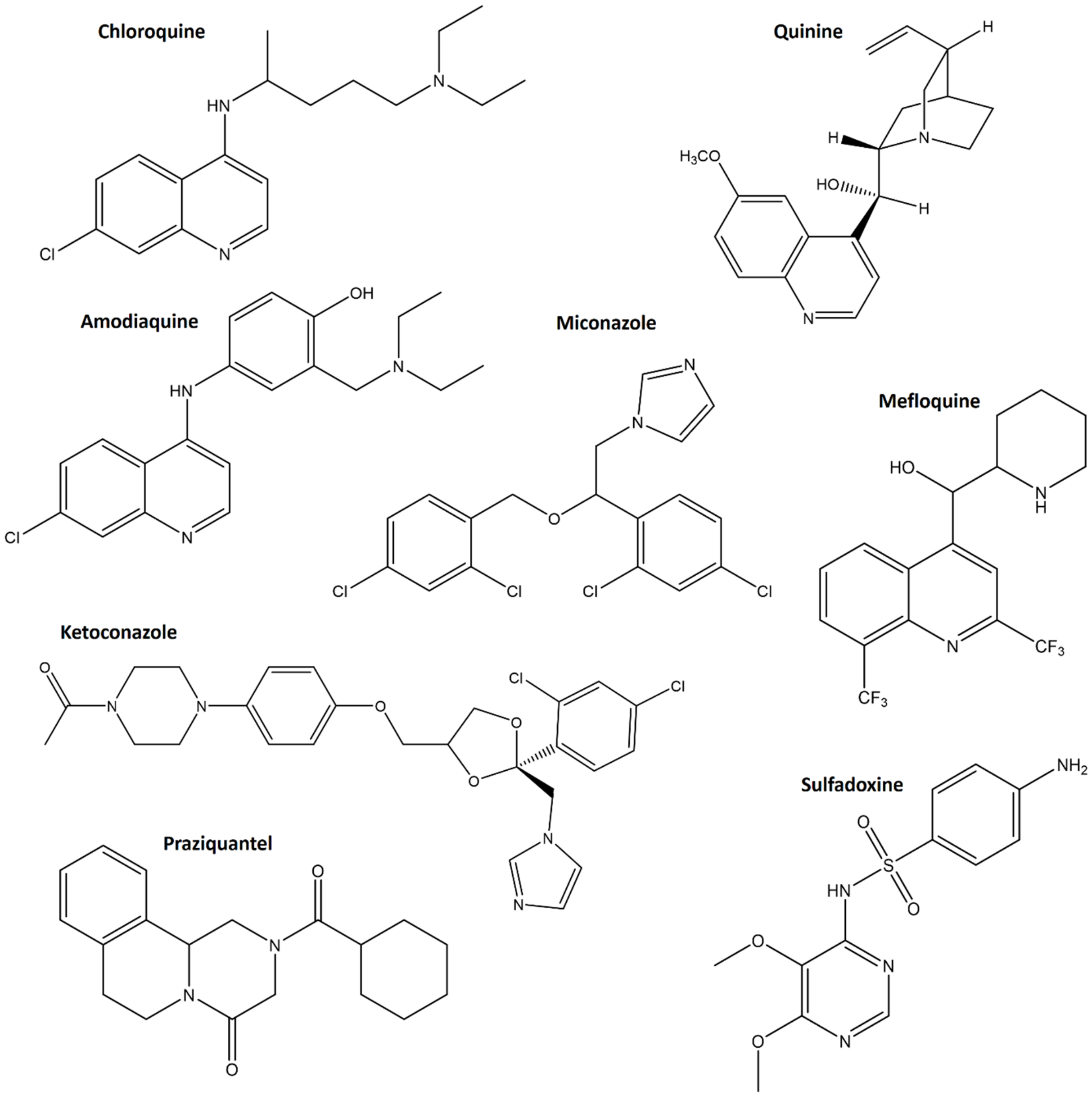

SI 12. Structures of alkaloids from *C. pubescens*

Figure S11. Structures of alkaloids from *C. pubescens*

cinchonidine

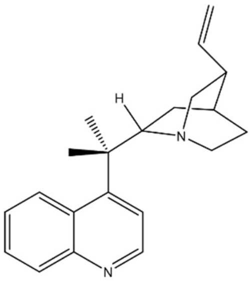

cinchonine

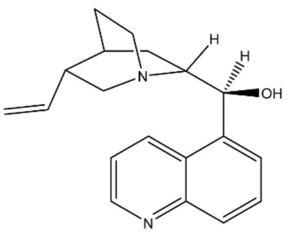

cupreine

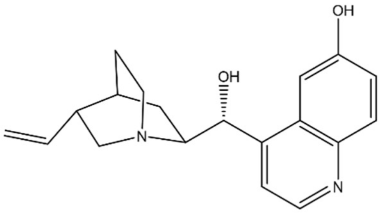

cupreidine

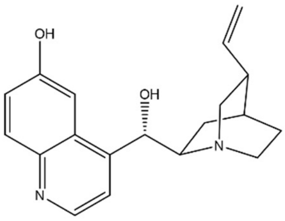

quinine

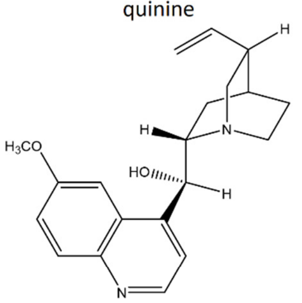

quinidine

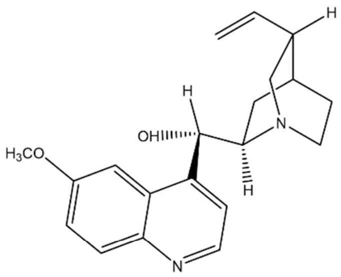

cinchonaminone

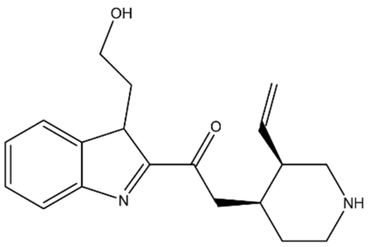

SI 13. References

1. Muñoz-Durango, K.; Maciuk, A.; Harfouche, A.; Torijano-Gutiérrez, S.; Jullian, J.-C.; Quintin, J.; Spelman, K.; Mouray, E.; Grellier, P.; Figadère, B. Detection, Characterization, and Screening of Heme-Binding Molecules by Mass Spectrometry for Malaria Drug Discovery. *Anal. Chem.* **2012**, *84*, 3324–3329, doi:10.1021/ac300065t.
2. Adityachaudhury, N.; Das, A.K.; Choudhury, A.; Daskanungo, P.L. Aurentiacin, a New Chalcone from *Didymocarpus Aurentiaca*. *Phytochemistry* **1976**, *15*, 229–230, doi:10.1016/S0031-9422(00)89099-8.
3. Resurreccion-Magno, Ma.H.C.; Villaseñor, I.M.; Harada, N.; Monde, K. Antihyperglycaemic Flavonoids from *Syzygium Samarangense* (Blume) Merr. and Perry. *Phytother. Res.* **2005**, *19*, 246–251, doi:10.1002/ptr.1658.
4. Harborne, J.B.; Baxter, H. *The Handbook of Natural Flavonoids. Volume 1 and Volume 2*; John Wiley and Sons, 1999; ISBN 978-0-471-95893-2.
5. Xiao, H.; Rao Ravu, R.; Tekwani, B.L.; Li, W.; Liu, W.-B.; Jacob, M.R.; Khan, S.I.; Cai, X.; Peng, C.-Y.; Khan, I.A.; et al. Biological Evaluation of Phytoconstituents from *Polygonum Hydropiper*. *Nat. Prod. Res.* **2017**, *31*, 2053–2057, doi:10.1080/14786419.2016.1269094.
6. Wollenweber, E.; Dietz, V.H.; Schilling, G.; Favre-Bonvin, J.; Smith, D.M. Flavonoids from Chemotypes of the Goldback Fern, *Pityrogramma Triangularis*. *Phytochemistry* **1985**, *24*, 965–971, doi:10.1016/S0031-9422(00)83163-5.
7. P., P.; Díaz, D.; C., T.A.; Joseph-Nathan, P. A Chromene an Isoprenylated Methyl Hydroxybenzoate and a C-Methyl Flavanone from the Bark of *Piper Hostmannianum*. *Phytochemistry* **1987**, *26*, 809–811, doi:10.1016/S0031-9422(00)84792-5.
8. Basnet, P.; Kadota, S.; Shimizu, M.; Xu, H.-X.; Namba, T. 2′-Hydroxymatteucinol, a New C-Methyl Flavanone Derivative from *Matteccia Orientalis*; Potent Hypoglycemic Activity in Streptozotocin (STZ)-Induced Diabetic Rat. *Chem. Pharm. Bull. (Tokyo)* **1993**, *41*, 1790–1795, doi:10.1248/cpb.41.1790.
9. Itokawa, H.; Morita, M.; Mihashi, S. Phenolic Compounds from the Rhizomes of *Alpinia Speciosa*. *Phytochemistry* **1981**, *20*, 2503–2506, doi:10.1016/0031-9422(81)83082-8.
10. Bick, I.; Brown, R.; Hillis, W. Three Flavanones from Leaves of *Eucalyptus Sieberi*. *Aust. J. Chem.* **1972**, *25*, 449–451.
11. Mayer, R. A *β*-Hydroxychalcone from Leptospermum Scoparium. *Planta Med* **2007**, *59*, 269–271, doi:10.1055/s-2006-959667.
12. Niu, X.M.; Li, S.H.; Peng, L.Y.; Lin, Z.W.; Rao, G.X.; Sun, H.D. Constituents from Limonia Crenulata. *J. Asian Nat. Prod. Res.* **2001**, *3*, 299–311, doi:10.1080/10286020108040370.
13. Chatterjee, A.; Chakrabarty, M.; Kundu, A. Constituents of Pleiospermium Alatum: Alatamide and N-Benzoyltyramine Methyl Ether. *Aust. J. Chem.* **1975**, *28*, 457–460.
14. Di Iorio, N.; Filippini, G.; Mazzanti, A.; Righi, P.; Bencivenni, G. Controlling the C(Sp3)–C(Sp2) Axial Conformation in the Enantioselective Friedel–Crafts-Type Alkylation of β-Naphthols with Inden-1-Ones. *Org. Lett.* **2017**, *19*, 6692–6695, doi:10.1021/acs.orglett.7b03415.
15. Szmant, H.H.; Nanjundiah, R. Thiol-Olefin Cooxidation Reaction. 6. A New Convenient Route to 1-Substituted Indenes. Indenone as Dienophile in Diels-Alder Reactions. *J. Org. Chem.* **1978**, *43*, 1835–1837, doi:10.1021/jo00403a056.
